# Biochemical and Computational Approach of Selected Phytocompounds from *Tinospora crispa* in the Management of COVID-19

**DOI:** 10.3390/molecules25173936

**Published:** 2020-08-28

**Authors:** Ahmed Rakib, Arkajyoti Paul, Md. Nazim Uddin Chy, Saad Ahmed Sami, Sumit Kumar Baral, Mohuya Majumder, Abu Montakim Tareq, Mohammad Nurul Amin, Asif Shahriar, Md. Zia Uddin, Mycal Dutta, Trina Ekawati Tallei, Talha Bin Emran, Jesus Simal-Gandara

**Affiliations:** 1 Department of Pharmacy, Faculty of Biological Sciences, University of Chittagong, Chittagong 4331, Bangladesh; rakib.pharmacy.cu@gmail.com (A.R.); s.a.sami18pharm@gmail.com (S.A.S.); 2 Drug Discovery, GUSTO A Research Group, Chittagong 4000, Bangladesh; arka.bgctub@gmail.com (A.P.); nazim107282@gmail.com (M.N.U.C.); mohuyamajumderbgctub@gmail.com (M.M.); 3 Department of Pharmacy, BGC Trust University Bangladesh, Chittagong 4381, Bangladesh; zia@bgctub.ac.bd (M.Z.U.); mycal@bgctub.ac.bd (M.D.); 4 Department of Microbiology, Jagannath University, Dhaka 1100, Bangladesh; akbaiub6@gmail.com; 5 Department of Pharmacy, International Islamic University Chittagong, Chittagong 4318, Bangladesh; montakim0.abu@gmail.com; 6 Department of Pharmacy, Atish Dipankar University of Science and Technology, Dhaka 1230, Bangladesh; amin.pharma07@gmail.com; 7 Department of Microbiology, Stamford University Bangladesh, 51 Siddeswari Road, Dhaka 1217, Bangladesh; abasifbl@gmail.com; 8 Department of Pharmacy, Jahangirnagar University, Savar, Dhaka 1342, Bangladesh; 9 Department of Biology, Faculty of Mathematics and Natural Sciences, Sam Ratulangi University, Manado 95115, Indonesia; trina_tallei@unsrat.ac.id; 10 Nutrition and Bromatology Group, Department of Analytical and Food Chemistry, Faculty of Food Science and Technology, University of Vigo–Ourense Campus, E32004 Ourense, Spain

**Keywords:** SARS-CoV-2, COVID-19, *Tinospora crispa*, natural products, phytochemicals, secondary metabolites, molecular docking

## Abstract

A pandemic caused by the novel coronavirus (SARS-CoV-2 or COVID-19) began in December 2019 in Wuhan, China, and the number of newly reported cases continues to increase. More than 19.7 million cases have been reported globally and about 728,000 have died as of this writing (10 August 2020). Recently, it has been confirmed that the SARS-CoV-2 main protease (M^pro)^ enzyme is responsible not only for viral reproduction but also impedes host immune responses. The M^pro^ provides a highly favorable pharmacological target for the discovery and design of inhibitors. Currently, no specific therapies are available, and investigations into the treatment of COVID-19 are lacking. Therefore, herein, we analyzed the bioactive phytocompounds isolated by gas chromatography–mass spectroscopy (GC-MS) from *Tinospora crispa* as potential COVID-19 M^pro^ inhibitors, using molecular docking study. Our analyses unveiled that the top nine hits might serve as potential anti-SARS-CoV-2 lead molecules, with three of them exerting biological activity and warranting further optimization and drug development to combat COVID-19.

## 1. Introduction

In late December 2019, Severe Acute Respiratory Disorder Coronavirus 2 (SARS-CoV-2) became an outbreak in China and then spread to other countries. Infected persons exhibit pneumonia indications, progressing to severe acute respiratory disorder. In January 2020, it was confirmed that an unknown sort of coronavirus named SARS-CoV-2 (formerly named as 2019-nCoV) had come out. On February 11, 2020, the World Health Organization (WHO) named the Wuhan pneumonia as Coronavirus Disease-2019 (COVID-19) and declared this infectious disease to be a global pandemic. Certain animals, including mammals, reptiles, and birds prone to this infectious disease caused by the virus are deadly, while there have been several deaths by respiratory contamination transmitted from animal to human then transmitted to humans [1,2].

The virus, known as coronavirus, a single RNA-stranded virus, belongs to the *Coronaviridae* family. The RNA is encapsulated in the membrane, and the virion contains phosphorylated nucleocapsid protein and RNA. By interacting with the angiotensin-converting-enzyme-2 (ACE2) receptor, the virus enters the host and causes severe respiratory tract infection (RTI) in humans. Viral proteases have been well documented as potential approved drug targets for several infections, including hepatitis C virus (HCV) and human immunodeficiency virus (HIV) [3]. Recent studies from Liu and his group documented the presence of the main protease (M^pro^) enzyme in SARS-CoV-2 [4]. Homology modeling with SARS-CoV M^pro^ has delineated about a 96% structural similarity with SARS-CoV M^pro^ [5]. Due to the structural similarity with the M^pro^ from other coronaviruses, SARS-CoV-2 M^pro^ can be considered to be a prospective target for drug discovery for COVID-19 treatment. SARS-CoV-2 M^pro^ cleaves the overlapping pp1a and pp1b polyproteins through proteolytic cleavage, which is considered to be a critical step for viral replication [6,7,8]. Additionally, the functioning of replication-essential enzymes, for instance, RNA-dependent RNA polymerase (RdRp), nsp13 are also dependent on the proteolytic release [9]. Consequently, SARS-CoV-2 M^pro^ is responsible for inhibiting SARS-CoV-2 replication. Therefore, targeting this specific enzyme will lead towards potential therapeutic advantages in drug discovery for this dangerous virus.

Scientists from all over the world are coming together and working simultaneously to fight against this deadly disease. However, so far, specific therapies regarding the treatment have been too little, and measures that have been implied are restricted to limited supportive and preventive therapies. Experimental trials have been carried out using a combination of lopinavir/ritonavir, which are commonly used to treat human immunodeficiency virus (HIV) infection [10]. Another experiment using four FDA approved drugs, including nelfinavir, pitavastatin, perampanel, and praziquantel, identified nelfinavir as a potent inhibitor for SARS-CoV-2 M^pro^ enzyme [11]. Although trials have already been carried out for the treatment of SARS-CoV-2 infection, preliminary investigations remain unapproved regarding their use.

Researchers have recently been searching for potent therapeutic agents from medicinal plants for SARS-CoV-2 infection [12]. Different secondary metabolites, including alkaloids, flavonoids, tannins, glycosides, lignin, and terpenes, are available from medicinal plants. Various studies have already established the function of phytochemicals in treating various infections and diseases [13,14,15,16,17,18]. Moreover, compound isolation and further investigation of several pharmacological activities have been established as a crucial factor in drug discovery [19].

In the present study, we tried to identify the role of some isolated phytochemicals from a medicinal plant, known as *Tinospora crispa* Miers., a member of the Menispermaceae family, for the treatment of COVID-19 using computational biology techniques. The plant is locally known as Gulancha [20]. It is indigenous to Bangladesh, Eastern China, India, and Malaysia. Our previous work also confirmed the hepatoprotective, antinociceptive, anxiolytic, antidepressant, and antipyretic activities of plant extract and fractions of *T. crispa* [21,22]. Moreover, the ethnobotanical role of the plant has already been documented, primarily as a tonic, a blood purifier, and for stomach disorders [20]. Importantly, the plant exhibited significant antimalarial attributes in combination with pyrimethamine, which corroborates the findings from recent studies providing evidence for the use of the antimalarial drug chloroquine and hydroxychloroquine to combat against SARS-CoV-2 [23,24,25]. In addition, one study reported the antiviral activity of *T. crispa* along with other traditional herbs delineated antiviral activity against fish pathogenic viruses, including infectious hematopoietic necrosis virus, infectious pancreatic necrosis virus, *Oncorhynchus masou* virus [26]. The active components from another species of *Tinospora* (*T. cordifolia*) has been shown to represent not only antiviral activity but also protease inhibitor activity [27]. Therefore, this study rationalizes the role of screened compounds of *T. crispa* to combat SARS-CoV-2.

## 2. Results

### 2.1. GC-MS Analysis

**Figure 1:**
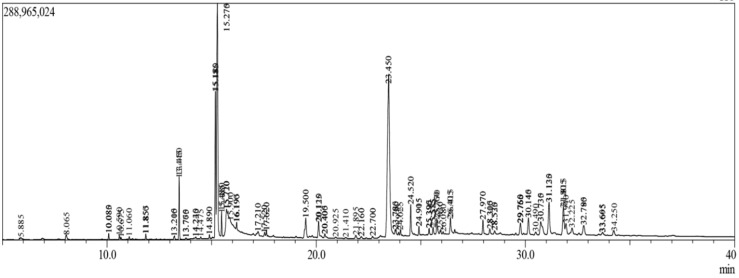
Total ionic chromatogram (TIC) of methanolic extract of *T. crispa* whole plants by GC-MS.

In 50 min retention time, the methanol extract of *T. crispa* contained a total of 309 compounds eluted between 5.0–40.0 min (Figure 1). Fifty-six (56) bioactive compounds were selected for this study (Table 1).

### 2.2. Prediction of Active Site

Using the CASTp server, we sought to identify the active pockets of the PDB protein. The area was depicted as 304.266, and the volume was 296.682. A total of 27 active site residues were identified by CASTp server, and the residues were Thr25, Thr26, Leu27, His41, Cys44, Thr45, Ser46, Met49, Pro52, Tyr54, Phe140, Leu141, Asn142, Gly143, Ser144, His163, His164, Met165, Glu166, Leu167, Pro168, His172, Asp187, Arg188, Gln189, Thr190, and Gln192.

### 2.3. Ligand-Based ADME/T Prediction

Lipinski’s rule of five was followed to predict the drug-likeliness properties; the following five principles must be followed: (i) Molecular weight is not more than 500; (ii) Number of H-bond acceptors ≤ 10; (iii) Number of H-bond donors ≤ 5; (iv) Lipophilicity (Log *P* value) < 5; and (v) Molar refractivity between 40 to 130. Among the fifty-six compounds, twenty-three compounds fulfilled the rule of five, indicating that those compounds could be suitable for the new drug development process. The results of the ADME/T prediction of the compounds is exhibited in Table 2.

### 2.4. Molecular Docking

**Figure 2:**
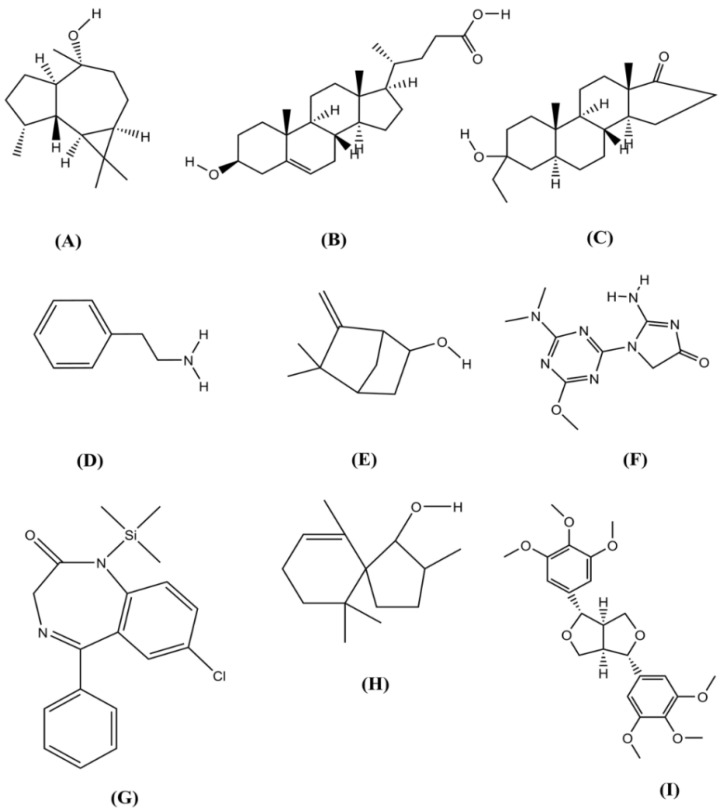
Chemical structure of (**A**) (−)-Globulol; (**B**) 3.beta-Hydroxy-5-cholen-24-oic acid; (**C**) Androstan-17-one, 3-ethyl-3-hydroxy-, 5; (**D**) Benzeneethanamine; (**E**) Camphenol; (**F**) Imidazolidin-4-one, 2-imino-1-(4-methoxy-6-dimethylamino-1,3,5-triazin-2-yl); (**G**) Nordazepam, TMS derivative; (**H**) Spiro[4,5]dec-6-en-1-ol, 2,6,10,10-tetramethyl; (**I**) Yangambin (structures are drawn using ChemDraw Professional version 16).

In this study, we are able to delineate the interaction between several isolated compounds of *T. crispa* with the M^pro^ enzyme of SARS-CoV-2 (PDB ID: 6W63). The docking results demonstrated that all compounds obtained from *T. crispa* interacted with the SARS-CoV-2 M^pro^ enzyme. Among these compounds, a total of seven possess higher docking scores in comparison with the others (Figure 2). However, some compounds exert a lower binding affinity towards the receptor (Table 3). 

**Figure 3:**
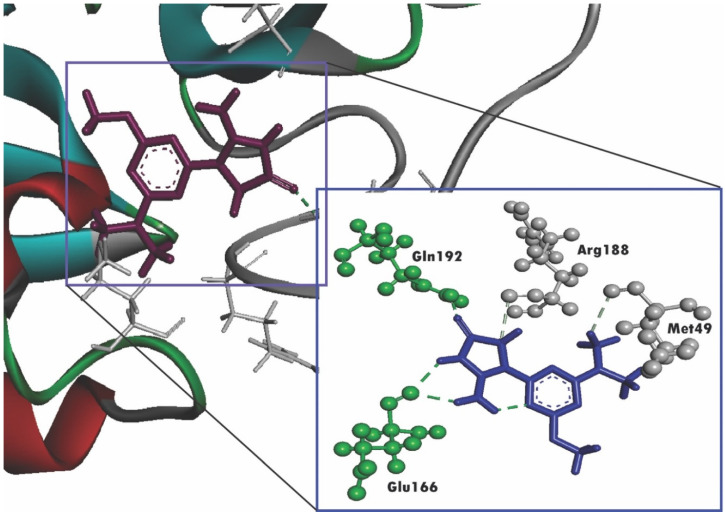
Molecular docking interaction between the SARS-CoV-2 M^pro^ and imidazolidin-4-one, 2-imino-1-(4-methoxy-6-dimethylamino-1,3,5-triazin-2-yl). The ligand in the active site is shown in purple color and the ligand interacting with the residues is shown in blue color, green color illustrates the residues forming hydrogen bonds, and white color illustrates the residues with carbon–hydrogen interaction.

Our computational investigation shows that imidazolidin-4-one, 2-imino-1-(4-methoxy-6-dimethylamino-1,3,5-triazin-2-yl) has the lowest docking score of −7.013 KJ/mol, interacting with Gly143 and Ser144 residues, respectively (Figure 3).

**Figure 4:**
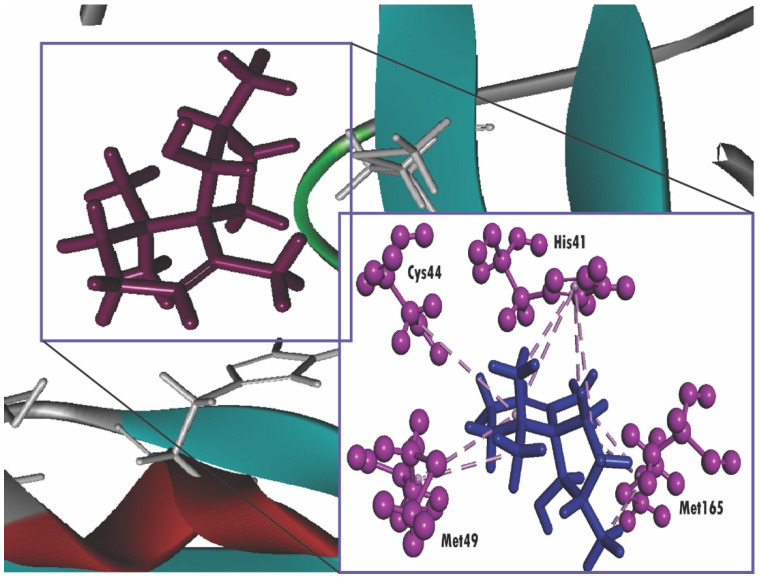
Molecular docking interaction between the SARS-CoV-2 M^pro^ and spiro[4,5]dec-6-en-1-ol, 2,6,10,10-tetramethyl. Ligand in the active site is shown in purple color, and ligand interacting with the residues are shown in blue color, pink color illustrated the residues with hydrophobic (pi-pi/pi-alkyl) stacking.

**Figure 5:**
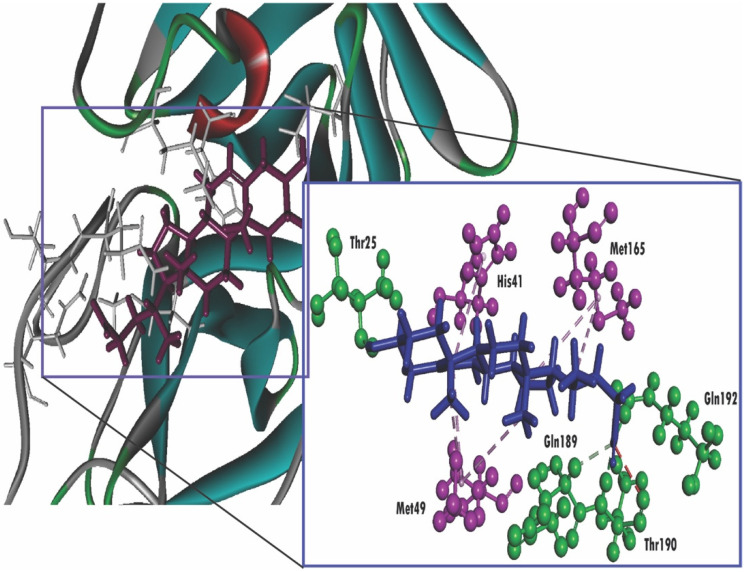
Molecular docking interaction between the SARS-CoV-2 M^pro^ and 3.beta-Hydroxy-5-cholen-24-oic acid ligand in the active site is shown in purple color, and ligand interacting with the residues is shown in blue color, green color illustrates the residues forming hydrogen bonds, pink color illustrates the residues with hydrophobic (pi-pi/pi-alkyl) stacking, and white color illustrates the residues with carbon-hydrogen interaction.

Additionally, spiro[4,5]dec-6-en-1-ol, 2,6,10,10-tetramethyl exhibits a docking score of −6.369 KJ/mol, but the interaction occurs with total four amino acids (Met165, His41, Met49, Met165) through pi-alkyl stacking, unlike imidazolidin-4-one, 2-imino-1-(4-methoxy-6-dimethylamino-1,3,5-triazin-2-yl), which interacts through hydrogen bonding (Figure 4). Moreover, 3.beta-Hydroxy-5-cholen-24-oic acid also tends to interact with the receptor as it has obtained a docking score of −6.251 KJ/mol. In addition, 3.beta-Hydroxy-5-cholen-24-oic acid possessed the affinity towards the receptor not only through hydrogen bonding but also pi-alkyl stacking (Figure 5). On the other hand, dibutyl phthalate (−2.279 kcal/mol) possessed the lowest score (Table 3). In particular, our experiment also includes the calculation of binding affinity of two antiviral agents, nelfinavir and lopinavir and these aforementioned compounds exert a score of −7.596 and −8.251 Kcal/mol, respectively, withSARS-CoV-2 M^pro^. The molecular docking analysis results are shown in Figure 3, Figure 4 and Figure 5, Figures S1–S13, and Table 3.

### 2.5. Prediction of Biological Activity

The selected compounds of the plant *T. crispa* were subjected to biological activity calculations with the help of Molinspiration software and compared with the standard drugs nelfinavir and lopinavir. The results are shown in Table 4.

## 3. Discussion

Currently, with an acute progression rate, it has been revealed that the clinical manifestations of SARS-CoV-2 infection start with a fever with a dry cough, which continues to alveolar edema, ultimately resulting in difficulty in breathing; however, mild symptoms might not include high fever [28,29,30]. Nevertheless, as the death counts are rising alarmingly, the respiratory infection triggered by SARS-CoV-2 has been classified as more critical in progressing the disease state in contrast with two other coronaviruses, SARS and MERS (Middle East Respiratory Syndrome), with a large number of infections and variations that spread rapidly while exhibiting minimal symptoms in the lungs. The organism can depreciate the normal functioning of the kidney, heart, liver, and other vital organs, leading to systemic exhaustion [31,32,33].

Although several kinds of research are being carried out in famous laboratories in several countries, vaccine development for disease is usually time-consuming. Despite there being plenty of experimental trials associated with COVID-19 treatment and medications, as of yet, scientists are still on the hunt for specific therapeutic drugs [34].

About 80% of certain Asian and African countries depend on traditional medicine for their major health care needs [35]. Several endogenous receptors responsible for prominent biological functions are triggered by numerous plant-derived phytochemicals [36]. Medicinal plants are endowed with plenty of phytocompounds, and since ancient times, plant-derived compounds have been used for treatment in numerous diseases [15,37,38]. Diverse secondary metabolites, including alkaloids, terpenoids, lignans, glycosides, amino acids, are crucial for the growth and functioning of plants and have numerous pharmacological aspects of fighting against several abnormal conditions [39,40,41]. Archaically, plant-derived chemicals were considered to be a prolific fount for drug discovery. Several previous studies have already documented the essentiality of phytochemicals in numerous diseases, including cardiovascular diseases, cancer, diabetes, hepatic disorders, etc. [42,43,44]. Many of these phytochemicals are generally not involved in the normal functioning of plants; nonetheless, they are modified by various biochemical processes and are further required for various environmental responses, including stress, protection from ultraviolet damage [45]. The extraction of phytochemicals has recently been established as a phenomenal subject matter in lead compound identification for active pharmaceutical moieties. More detailed information about various pharmacologically active medicinal plants’ crude extracts could be obtained using separation techniques, which involves the separation of active phytoconstituents [46,47]. In this experiment, we used the GC-MS technique for qualitative analysis of the plant extracts of *T. crispa* and confirmed more than 300 compounds.

Recently, researchers from different parts of the world have been working extensively to find certain potential lead compounds from medicinal plants that are active against several enzymes and other proteins responsible for viral replication and growth [33,48,49]. In line with this, we planned in silico experiments using the isolated constituents from methanol extract of *T. crispa* against the SARS-CoV-2 M^pro^ enzyme. Significantly, we have already mentioned the role of *T. crispa* extracts against malaria and the recent successful usage of anti-malarial drugs in combatting COVID-19, which leads the foundation for our hypothesis.

Nowadays, drug design using various bioinformatics tools has been proven as groundbreaking methods in drug discovery not only due to promptness and accuracy but also its low cost [50]. Molecular docking simulation, a form of bioinformatics analysis, signifies the binding affinities of ligand molecules with a specific receptor, in which the lower binding energy predicts the higher binding affinity [51,52,53]. A recent study from Yamamoto et al. has already shown that nelfinavir can inhibit SARS-CoV-2 replication in vitro [54]. Previously, another study from Yamamoto et al. reported the inhibitory effect of nelfinavir on the replication of SARS-CoV [55]. In addition, another in vitro analysis showed that lopinavir/ritonavir exhibited potential inhibitory effects on SARS-CoV-2 [56]. Hence, we selected nelfinavir and lopinavir as positive controls in the present study. In this study, our selected phytocompounds, along with two antiviral drugs, nelfinavir and lopinavir, were able to dock with the active pockets of SARS-CoV-2 M^pro^ enzyme, which was confirmed by our analysis through CASTp web server. Despite showing lower binding affinities than nelfinavir and lopinavir, our selected compounds interacted with the active pockets of the SARS-CoV-2 M^pro^ enzyme like the standard compounds. Additionally, imidazolidin-4-one, 2-imino-1-(4-methoxy-6-dimethylamino-1,3,5-triazin-2-yl) not only has a docking score almost the same as nelfinavir, but also, like nelfinavir, interacts with Glu166 residue through H-bonding. On the other hand, the interactions between spiro[4,5]dec-6-en-1-ol, 2,6,10,10-tetramethyl and 3.beta-hydroxy-5-cholen-24-oic acid with SARS-CoV-2 M^pro^ enzyme were found to be more than both the standard drugs. Additionally, other compounds, including androstan-17-one, 3-ethyl-3-hydroxy-, (5.alpha), camphenol, (−)-Globulol, yangambin, nordazepam, TMS derivative, and benzeneethanamine, also represented greater interaction with the abovementioned enzyme. Previously, it was found that the His41 and Cys145 residues belong to the catalytic dyads of the SARS-CoV-2 M^pro^ [57]. In the current study, the results of the docking analysis revealed that retinal, retinol, spiro[4,5]dec-6-en-1-ol, 2,6,10,10-tetramethyl, phosphonoacetic Acid, 3TMS derivative, aR-turmerone, androstan-17-one, 3-ethyl-3-hydroxy-, (5.alpha) interacted with Cys145 residue through hydrophobic interaction. Furthermore, the two standard drugs, along with most of the selected compounds, interacted with His41 residue. Like nelfinavir, benzeneethanamine also yielded interaction with His41 residue by forming hydrogen bonds. Although neither nelfinavir nor lopinavir interacted with Met49 residues, most of the targeted compounds interacted with Met49 residues, and this residue, along with His41, is crucial for substrate-binding [57]. In addition, Gly143, Ser144, His163, His164, Met165, Glu166, Leu167, Asp187, Arg188, Gln189, Thr190, Ala191, and Gln192 residues are also crucial for substrate binding in SARS-CoV-2 M^pro^ [58,59]. Our analysis showed that 3,4-dihydroxymandelic interacted with His163, Ser144 from the substrate-binding domain through hydrogen bonding. In addition, camphenol, possessing a greater docking score, formed a hydrogen bond with substrate-binding His164 residue. Moreover, most of the selected compounds as well as nelfinavir interacted with Met165 residue by forming a hydrophobic interaction.

Moreover, the selected compounds also followed Lipinski’s rule of five for drug-likeness properties. Furthermore, the ion channel inhibitor property of spiro[4,5]dec-6-en-1-ol, 2,6,10,10-tetramethyl were found more than standard compounds. Additionally, imidazolidin-4-one, 2-imino-1-(4-methoxy-6-dimethylamino-1,3,5-triazin-2-yl) possessed closer kinase inhibitor attributes in comparison with the standards. In addition, 3.beta-Hydroxy-5-cholen-24-oic acid exerted greater nuclear receptor ligand than nelfinavir and lopinavir. Interestingly, both spiro[4,5]dec-6-en-1-ol, 2,6,10,10-tetramethyl, 3.beta-Hydroxy-5-cholen-24-oic acid and 3.beta-Hydroxy-5-cholen-24-oic acid presented greater enzyme inhibition than either antiviral drug, whereas imidazolidin-4-one, 2-imino-1-(4-methoxy-6-dimethylamino-1,3,5-triazin-2-yl) exhibited greater protease blocking activity.

## 4. Materials and Methods

### 4.1. Plant Collection

The whole plant of *T. crispa* was collected at the mature stage from the Lawachara National Park, Moulavi Bazar, Bangladesh, in January 2018. The plant parts were cut into small pieces that were washed under tap water and then dried in the dark at 21–30 °C for 15 days. The whole plant material was ground by a mechanical grinder and passed through a size of 60 mesh sieve to obtain a fine powder that was stored in an air-tight container.

### 4.2. Preparation of Extracts

The dried *T. crispa* plant powder (600 g) was macerated in 4 L methanol (Merck, Darmstadt, Germany) for 15 days at room temperature with occasional shaking and stirring. Following filtration, first with a cotton plug, then with a Whatman No. 1 filter paper, the filtrate was evaporated to dryness under vacuum at 40 °C to obtain a concentrated extract (30.55 g dry weight, 5.09% *w*/*w*). The extract was preserved for further analysis.

### 4.3. Gas Chromatography–Mass Spectroscopy (GC-MS) Analysis

The GC-MS analysis was evaluated using a model 7890A capillary gas chromatography along with a mass spectrometer (Agilent Technologies, Santa Clara, CA, USA). The column was a fused silica capillary column of 95% dimethyl-poly siloxane and 5% phenyl (HP-5MSI; length: 90 m, diameter: 0.250 mm and film: 0.25 µm, Merck, Darmstadt, Germany). Parameters for GC-MS detection were an injector temperature of 250 °C, and the initial oven temperature of 90 °C was gradually raised to 200 °C at a speed of 3 °C/min for 2 min and with a final increase to 280 °C at 15 °C/min for 2 min. The total GC-MS run time was 36 min, using 99.999% helium as a carrier gas, at a column flow rate of 1 mL/min. The GC to MS interface temperature was fixed at 280 °C, and an electron ionization system was set on the MS in scan mode. The mass range evaluated was 50–550 *m*/*z*, where MS quad and source temperatures were maintained at 150 °C and 230 °C, respectively. The NIST-MS Library 2009 was used to search and identify each component, and to measure the relative percentage of each compound, relative peak areas of the TIC (total ionic chromatogram) were used, with calculations performed automatically.

### 4.4. Active Site Prediction

Potential ligand binding sites/pockets (active sites) on the 3D structure of protein were identified by the CASTp web server (http://sts.bioe.uic.edu/castp/) [60]. CASTp uses the recent algorithmic and geometrical analysis of computational chemistry for the analytical validation pockets and cavities.

### 4.5. In Silico ADME Analysis

The pharmacokinetic properties of all major identified compounds were evaluated using Lipinski’s rule of five [61]. Lipinski stated that a compound could show drug-like behavior if it does not fail more than one of the following criteria: (i) Molecular weight is not more than 500; (ii) H-bond donors ≤ 5; (iii) H-bond acceptors ≤ 10; (iv) Lipophilicity < 5; and (v) Molar refractivity between 40 and 130. The web tool Swiss ADME was used to assess the ADME parameters of all compounds. Compounds obeying the Lipinski rule are considered as ideal drug candidates [62].

### 4.6. Computational Molecular Docking Analysis

#### 4.6.1. Ligand Preparation

The selected isolated compounds of *T. crispa* were subjected to Maestro v 10.1 (Schrödinger suite, LLC New York, NY, USA), and ligand preparation was done using the LigPrep tool. The parameters were set to neutralize at pH 7.0 ± 2.0 using Epik 2.2, and minimized by force field OPLS_2005.

#### 4.6.2. Protein Preparation

3D crystal structure of SARS-CoV-2 M^pro^ (PDB ID: 6W63) was downloaded from the Protein Data Bank and prepared using the protein preparation wizard of the Schrödinger Suite—Maestro version 10.1. Charges and bond orders were assigned, hydrogens added to heavy atoms and selenomethionines and selenocysteines converted into methionines and cysteines, respectively, followed by removing all water molecules. Using force field OPLS_2005, minimization was performed to set a maximum heavy atom RMSD to 0.30 Å.

#### 4.6.3. Receptor Grid Generation and Molecular Docking

Receptor grid generation and molecular docking experiments were performed using Glide (Schrödinger Suite—Maestro version 10.1) [63,64]. A grid was produced for each protein using the following default parameters: van der Waals scaling factor 1.00 and charge cut-off value 0.25, subjected to the OPLS_2005 force field. A cubic box of definite dimensions centered on the centroid of the active site residues was generated for the receptor, and the box size was set to 14 Å × 14 Å × 14 Å for docking. Docking experiments were carried out using the standard precision (SP) scoring function of glide, and only the best scoring fit with docking score was noted for each ligand.

### 4.7. Biological Activity Prediction

The targeted compounds were assessed for potential bioactivity by calculating their activity scores as GPCR ligands, ion channel modulators, kinase inhibitors, nuclear receptor inhibitors, and enzyme inhibitors. All the parameters were checked with the aid of the software Molinspiration (www.molinspiration.com, Nova Ulica, Slovensky Grob, Slovak Republic) [21]. Calculated drug-likeness scores of each compound were compared with each compound’s specific activity and were compared with the standard drugs (nelfinavir and lopinavir).

## 5. Conclusions

COVID-19 created a devastating global crisis impacting thousands of people every day, taking thousands of lives and hampering the global economy. Virtual molecular docking was conducted to identify new compounds that could bind the SARS-CoV-2 M^pro^. The isolated compounds obtained from the methanol extract of *T. crispa* were investigated in silico, which concluded that some selective compounds are potentially enough to alter the activity of the SARS-CoV-2 M^pro^ enzyme. Our analysis indicates phytochemicals from the methanolic extract of *T. crispa* such as imidazolidin-4-ne, 2-imino-1-(4-methoxy-6-dimethylamino-1,3,5-triazin-2-yl), spiro[4,5]dec-6-en-1-ol, 2,6,10,10-tetramethyl, 3.beta-hydroxy-5-cholen-24-oic acid, androstan-17-one, 3-ethyl-3-hydroxy-(5.alpha), camphenol, (−)-Globulol, yangambin, nordazepam, TMS derivative, benzeneethanamine have a better binding affinity to M^pro^ of SARS-CoV-2 compared to nelfinavir and lopinavir. Further research and development will pave the way for identifying possible SARS-CoV-2 M^pro^ inhibitors. We anticipate that the insights obtained in this study can be useful for the potential discovery and development of new natural anti-COVID-19 therapeutic agents.

## Figures and Tables

**Table 1:** Quantitative compounds identified from methanol extract of *T. crispa* by GC-MS analysis.

Compound Name	Retention Time	*m*/*z*	Area	PA (%)
Benzeneethanamine	10.083	73.00	3196250	0.106518
Camphenol	13.761	107.00	431753	0.014389
Strophanthidin	22.159	79.00	334706	0.011154
Retinal	22.159	79.00	334706	0.011154
Trans-Geranylgeraniol	24.911	69.00	4724235	0.15744
3,4-Dihydroxymandelic acid	17.261	73.00	2174852	0.072479
Imidazolidin-4-one, 2-imino-1-(4-methoxy-6-dimethylamino-1,3,5-triazin-2-yl)	25.586	251.00	4374165	0.145774
Cholest-22-ene-21-ol, 3,5-dehydro-6-methoxy	25.771	95.00	5058177	0.168569
d-Mannitol, 1-O-(16-hydroxyhexadecyl)-	26.431	207.00	153759	0.005124
Heneicosanoic acid, methyl ester	26.431	207.00	153759	0.005124
Gorgost-5-en-3-ol, (3.beta)-, TMS derivative	26.431	207.00	153759	0.005124
Retinol	26.414	91.00	1957701	0.065242
Octacosanol	27.969	97.00	3033614	0.101098
Alpha-Santalol	28.532	94.00	3133161	0.104416
Santalol, E-cis,epi-beta-	28.532	94.00	3133161	0.104416
Spiro[4,5]dec-6-en-1-ol, 2,6,10,10-tetramethyl	28.532	94.00	3133161	0.104416
Campesterol	29.551	207.00	243053	0.0081
Cholesterol	29.551	207.00	243053	0.0081
9,19-Cyclocholestan-3-ol, 14-methyl-, (3.beta)	29.551	207.00	243053	0.0081
Cholest-5-en-3-ol, 6-methyl-, (3.beta)-	29.551	207.00	243053	0.0081
26-Hydroxycholesterol	29.551	207.00	243053	0.0081
Beta-Sitosterol	29.551	207.00	243053	0.0081
Lathosterol	29.551	207.00	243053	0.0081
Ergost-7-en-3-ol	29.551	207.00	243053	0.0081
Cholest-5-en-3-ol (3.beta)-, carbonochloridate	29.551	207.00	243053	0.0081
Stigmasterol	29.551	207.00	243053	0.0081
Cholesta-5,22-dien-3-ol, (3.beta)-	29.551	207.00	243053	0.0081
Ergosta-5,24(28)-dien-3-ol, (3.beta)-	29.551	207.00	243053	0.0081
Lathosterol	29.551	207.00	243053	0.0081
Cholestane-3,5-diol, 5-acetate, (3.beta,5.alpha	29.551	207.00	243053	0.0081
26,27-Dinorergosta-5,23-dien-3-ol, (3.beta)-	30.145	55.00	2543634	0.084769
Desmosterol	30.145	55.00	2543634	0.084769
5,6-Dihydroergosterol	30.145	55.00	2543634	0.084769
9,19-Cyclolanost-23-ene-3,25-diol, 3-acetate	30.787	207.00	142424	0.004746
Lupeol	30.787	207.00	142424	0.004746
3.beta-Hydroxy-5-cholen-24-oic acid	31.132	43.00	4086523	0.136188
26,27-Dinorergost-5-ene-3,24-diol, (3.beta)-	31.132	43.00	4086523	0.136188
9,19-Cyclolanostan-3-ol, 24-methylene-, (3.beta)	29.551	95.00	3611829	0.120368
Lupeol, trifluoroacetate	31.820	95.00	3611829	0.120368
Lup-20(29)-en-3-ol, acetate, (3beta)-	32.780	207.00	1038162	0.034598
Phosphonoacetic Acid, 3TMS derivative	5.819	73.00	653645	0.021783
Nordazepam, TMS derivative	5.819	73.00	653645	0.021783
2,6-Dihydroxybenzoic acid, 3TMS derivative	10.083	73.00	3196250	0.106518
aR-Turmerone	10.592	83.00	2528243	0.084256
(Z)-.gamma.-Atlantone	10.592	83.00	2528243	0.084256
Verbenylangelate, cis-	10.592	83.00	2528243	0.084256
Tumerone	10.592	83.00	2548801	0.084941
Dibutyl phthalate	13.793	149.00	1573158	0.052427
(−)-Globulol	22.159	79.00	334706	0.011154
Androstan-17-one, 3-ethyl-3-hydroxy-, (5.alpha)	22.159	79.00	334706	0.011154
Eudesma-4(15),7-dien-1.beta –ol	22.159	79.00	334706	0.011154
5.alpha-Cholest-8-en-3-one, 14-methyl-	25.771	95.00	5058177	0.168569
25-Hydroxycholesterol, 3-methyl ether	26.622	207.00	362457	0.012079
26-Homo-25-hydroxycholesterol	29.551	207.00	243053	0.0081
Betulin	32.780	207.00	1038162	0.034598
Yangambin	34.225	207.00	1108879	0.036955

*m*/*z*: m stands for mass and *z* stands for the charge number of ions, PA: Peak Area.

**Table 2:** ADME properties of selected compounds methanol extract of *T. crispa* by SwissADME.

Compound Name	Molecular Weight	Num. H-Bond Acceptors	Num. H-Bond Donors	Log *P*	Molar Refractivity	No. of Violation
Benzeneethanamine	121.18	1	1	1.7	38.92	0
Camphenol	152.23	1	1	2.30	46.38	0
Strophanthidin	404.5	6	3	1.82	106.16	0
Retinal	284.44	1	0	4.39	93.71	0
Trans-Geranylgeraniol	290.48	1	1	4.95	97.52	0
3,4-Dihydroxymandelic acid	184.15	5	4	−0.36	43.19	0
Imidazolidin-4-one, 2-imino-1-(4-methoxy-6-dimethylamino-1,3,5-triazin-2-yl)	251.25	6	1	−0.59	70.28	0
Cholest-22-ene-21-ol, 3,5-dehydro-6-methoxy	498.78	3	0	6.39	151.03	1
d-Mannitol, 1-*O*-(16-hydroxyhexadecyl)-	422.60	7	6	0.74	115.92	1
Heneicosanoic acid, methyl ester	340.58	2	0	5.58	109.15	1
Gorgost-5-en-3-ol, (3.beta)-, TMS derivative	498.90	1	0	7.49	157.87	2
Retinol	286.45	1	1	4.48	94.67	0
Octacosanol	410.76	1	1	7.07	137.87	2
Alpha-Santalol	220.35	1	1	3.67	68.04	0
Santalol, E-cis,epi-.beta-	220.35	1	1	3.56	69.94	0
Spiro[4,5]dec-6-en-1-ol, 2,6,10,10-tetramethyl	208.34	1	1	3.41	65.35	0
Campesterol	400.68	1	1	6.54	128.42	1
Cholesterol	386.65	1	1	6.34	123.61	1
9,19-Cyclocholestan-3-ol, 14-methyl-, (3.beta)	400.68	1	1	6.68	126.26	1
Cholest-5-en-3-ol, 6-methyl-, (3.beta)-	400.68	1	1	6.54	128.42	1
26-Hydroxycholesterol	402.65	2	2	5.41	124.78	1
Beta-Sitosterol	414.71	1	1	6.73	133.23	2
Lathosterol	386.65	1	1	6.34	123.61	1
Ergost-7-en-3-ol	400.68	1	1	6.54	128.42	1
Cholest-5-en-3-ol (3.beta)-, carbonochloridate	449.11	2	0	6.51	133.73	2
Stigmasterol	412.69	1	1	6.62	132.75	2
Cholesta-5,22-dien-3-ol, (3.beta)-	384.64	1	1	6.23	123.14	1
Ergosta-5,24(28)-dien-3-ol, (3.beta)-	398.66	1	1	6.43	127.95	1
Lathosterol	386.65	1	1	6.34	123.61	1
Cholestane-3,5-diol, 5-acetate, (3.beta,5.alpha	446.71	3	1	5.74	135.03	2
26,27-Dinorergosta-5,23-dien-3-ol, (3.beta)-	370.61	1	1	6.03	118.33	1
Desmosterol	384.64	1	1	6.23	123.14	1
5,6-Dihydroergosterol	398.66	1	1	6.43	127.95	1
9,19-Cyclolanost-23-ene-3,25-diol, 3-acetate	484.75	3	1	6.20	146.08	2
Lupeol	426.72	1	1	6.92	135.14	2
3.beta-Hydroxy-5-cholen-24-oic acid	374.56	3	2	4.62	110.97	0
26,27-Dinorergost-5-ene-3,24-diol, (3.beta)-	388.63	2	2	5.21	120.01	1
9,19-Cyclolanostan-3-ol, 24-methylene-, (3.beta)	440.74	1	1	7.12	139.95	2
Lupeol, trifluoroacetate	522.73	5	0	7.36	145.07	3
Lup-20(29)-en-3-ol, acetate, (3beta)-	468.75	2	0	7.08	144,.88	2
Phosphonoacetic Acid, 3TMS derivative	356.58	5	0	1.42	90.71	0
Nordazepam, TMS derivative	342.89	2	0	3.14	105.43	0
2,6-Dihydroxybenzoic acid, 3TMS derivative	370.66	4	0	2.97	103.15	0
aR-Turmerone	216.32	1	0	3.68	69.75	0
(*Z*)-gamma.-Atlantone	218.33	1	0	3.37	70.88	0
Verbenylangelate, cis-	234.33	2	0	3.35	70.07	0
Tumerone	218.33	1	0	3.37	70.88	0
Dibutyl phthalate	278.34	4	0	3.43	77.84	0
(−)-Globulol	222.37	1	1	3.81	68.82	0
Androstan-17-one, 3-ethyl-3-hydroxy-,(5.alpha)	318.49	2	1	4.15	95.48	0
Eudesma-4(15),7-dien-1.beta–ol	220.35	1	1	3.56	69.94	0
5.alpha-Cholest-8-en-3-one, 14-methyl-	398.66	1	0	6.43	127.20	1

**Table 3:** Molecular docking study of major bioactive compounds of methanol extract of *T. crispa.*

Compound Name	Docking Score	Residues Interacting with Ligand through H-Bonding (H-Bonds No.)	Hydrophobic Bonds (pi-Alkyl Stacked)	Hydrophobic Bonds (pi-pi Stacked)
Camphenol	−6.177	His164		His41, Met49 (3), Met165
Strophanthidin	−5.8	Gln189		Met165
Benzeneethanamine	−6.022	His41	His41	
Retinal	−5.591	His163	His41, Cys145,Cys44, Met49	
Trans-geranylgeraniol	−3.393	His163, Ser144	Met49 (2), Arg188	
3,4-Dihydroxymandelic acid	−5.51	Gly143,Ser144		
Imidazolidin-4-one, 2-imino-1-(4-methoxy-6-dimethylamino-1,3,5-triazin-2-yl)	−7.013	Glu166 (2), Gln192		
Retinol	−5.576	Thr24	Cys145, Met165, His41, Cys44, Met49	
Alpha-Santalol	−5.595	Gln192	His41 (2), Met49 (3), Pro168, Met165	
Santalol, E-cis, epi-beta-	−5.664	Gln192	His41, Met49 (2), Pro168,	
Spiro[4,5]dec-6-en-1-ol, 2,6,10,10-tetramethyl	−6.369		Met165 (3), His41 (4), Met49 (2), Cys145	
3.beta-Hydroxy-5-cholen-24-oic acid	−6.251	Thr25, Thr190,Gln192	Met49 (3), His41, Met165 (2)	
Phosphonoacetic Acid, 3TMS derivative	−4.273	Glu166	Cys145 (2), His41 (2), Met165, His163	
Nordazepam, TMS derivative	−6.122	Glu166	Met165	
2,6-Dihydroxybenzoic acid, 3TMS derivative	−4.696		Met165 (2), His41 (3), Cys145	
aR-Turmerone	−5.452	Cys44	Met165, Pro168, His41 (2), Met49	
(Z)-gamma.-Atlantone	−5.708	Gln189	Met49, Met165, His41, Pro168 (2)	
Verbenyl angelate, cis-	−5.579		Met165 (2), Pro52, Arg188, Cys44 (4), Met49 (4)	
Tumerone	−5.131		Met165 (2), His41 (2), Cys44	
Dibutyl phthalate	−2.279	Gly143, Asn142	His41, His163, His172	
(−)-Globulol	−6.165			Met49 (2), His41(4), Cys44 (2), Met165 (3)
Androstan-17-one, 3-ethyl-3-hydroxy-(5.alpha)	−6.218	Thr26	Cys145, Met49, His41 (3)	
Yangambin	−6.162	Thr25	Met49, Met165	His41
Nelfinavir	−7.596	His41, Glu166 (2)	His41	Met165
Lopinavir	−8.251	Gln189, Glu166, Cys141, Thr26	Pro168, His41 (2)	Arg188, His41

**Table 4:** Biological activity prediction of methanol extract of *T. crispa.*

Compounds	GPCR Ligand	Ion Channel Inhibitor	Kinase Inhibitor	Nuclear Receptor Ligand	Protease Inhibitor	Enzyme Inhibitor
Benzeneethanamine	−1.71	−1.16	−1.95	−2.61	−1.85	−1.43
Camphenol	−0.66	−0.43	−1.53	−0.62	−1.06	−0.37
Strophanthidin	0.08	0.07	−0.46	0.52	0.01	0.79
Retinal	−0.15	0.15	−0.23	0.90	0.09	0.52
Trans-Geranylgeraniol	0.12	0.20	−0.22	0.40	−0.08	0.41
3,4-Dihydroxymandelic acid	−0.28	−0.18	−0.69	−0.06	−0.61	−0.05
Imidazolidin-4-one, 2-imino-1-(4-methoxy-6-dimethylamino-1,3,5-triazin-2-yl)	0.15	−0.15	−0.20	−0.63	−0.55	0.08
Retinol	−0.01	0.32	−0.25	1.02	−0.16	0.66
Alpha-Santalol	−0.04	0.03	−0.43	0.12	−0.22	0.37
Santalol, *E*-cis,epi-.beta-	−0.09	−0.04	−0.65	0.23	−0.42	0.39
Spiro[4,5]dec-6-en-1-ol, 2,6,10,10-tetramethyl	−0.23	0.00	−0.79	0.40	−0.27	0.43
3.beta-Hydroxy-5-cholen-24-oic acid	0.20	0.03	−0.57	0.87	0.07	0.64
Phosphonoacetic Acid, 3TMS derivative	0.43	0.59	0.12	−0.05	0.71	1.08
Nordazepam, TMS derivative	0.48	0.58	−0.22	−0.07	0.33	0.35
2,6-Dihydroxybenzoic acid, 3TMS derivative	0.34	0.12	−0.03	−0.06	0.42	0.72
aR-Turmerone	−0.68	−0.46	−1.36	−0.14	−0.80	−0.25
(*Z*)-gamma.-Atlantone	−0.38	0.15	−1.17	0.37	−0.58	0.46
Verbenyl angelate, cis-	−0.09	−0.08	−0.98	0.32	−0.35	0.28
Tumerone	−0.35	−0.13	−1.19	0.54	−0.44	0.41
Dibutyl phthalate	−0.16	−0.09	−0.27	−0.12	−0.25	−0.07
(−)-Globulol	−0.50	−0.29	−0.82	−0.22	−0.48	−0.13
Androstan-17-one, 3-ethyl-3-hydroxy-, (5.alpha)	0.19	0.41	−0.35	0.83	0.17	0.66
Yangambin	−0.03	−0.25	−0.19	−0.10	−0.16	0.01
Nelfinavir	0.19	−0.25	−0.28	−0.25	0.58	−0.02
Lopinavir	0.04	−0.78	−0.55	−0.66	0.42	−0.37

## Supplementary Materials

The following are available online. Figure S1: 2D and 3D interactions of Benzeneethanamine (A) and Camphenol (B) with the active site of SARS-CoV-2 M^pro^ (PDB ID: 6W63); Figure S2: 2D and 3D interactions of Strophanthidin (C) and Retinal (D) with the active site of SARS-CoV-2 M^pro^ (PDB ID: 6W63); Figure S3: 2D and 3D interactions of Trans-geranylgeraniol (E) and 3,4-Dihydroxymandelic acid (F) with the active site of SARS-CoV-2 M^pro^ (PDB ID: 6W63); Figure S4. 2D and 3D interactions of Imidazolidin-4-one, 2-imino-1-(4-methoxy-6-dimethylamino-1,3,5-triazin-2-yl) (G) and Retinol (F) with the active site of SARS-CoV-2 M^pro^ (PDB ID: 6W63); Figure S5: 2D and 3D interactions of alpha-Santalol (I) and Santalol, E-cis,epi-.beta- (J) with the active site of SARS-CoV-2 M^pro^ (PDB ID: 6W63); Figure S6: 2D and 3D interactions of Spiro[4,5]dec-6-en-1-ol, 2,6,10,10-tetramethyl (K) and 3.beta-Hydroxy-5-cholen-24-oic acid (L) with the active site of SARS-CoV-2 M^pro^ (PDB ID: 6W63); Figure S7: 2D and 3D interactions of Phosphonoacetic acid, 3TMS derivative (M) and Nordazepam, TMS derivative (N) with the active site of SARS-CoV-2 M^pro^ (PDB ID: 6W63); Figure S8: 2D and 3D interactions of 2,6-Dihydroxybenzoic acid, 3TMS derivative (O) and aR-Turmerone (P) with the active site of SARS-CoV-2 M^pro^ (PDB ID: 6W63); Figure S9: 2D and 3D interactions of (Z)-.gamma.-Atlantone (Q) and Verbenyl angelate, cis- (R) with the active site of SARS-CoV-2 M^pro^ (PDB ID: 6W63); Figure S10. 2D and 3D interactions of Tumerone (S) and Dibutyl phthalate (T) with the active site of SARS-CoV-2 M^pro^ (PDB ID: 6W63); Figure S11: 2D and 3D interactions of (-)-Globulol (U) and Androstan-17-one, 3-ethyl-3-hydroxy-, (5.alpha) (V) with the active site of SARS-CoV-2 M^pro^ (PDB ID: 6W63); Figure S12: 2D and 3D interactions of Yangambin (W) with the active site of SARS-CoV-2 M^pro^ (PDB ID: 6W63); Figure S13: 2D and 3D interactions of Nelfinavir (X) and Lopinavir (Y) with the active site of SARS-CoV-2 M^pro^ (PDB ID: 6W63).
