# Supplementary material for: Biochemical and Computational Approach of Selected Phytocompounds from Tinospora crispa in the Management of COVID-19
Source: Molecules. 2020 Aug 28;25(17):3936. doi: 10.3390/molecules25173936 (PMC7504753; doi:10.3390/molecules25173936)
Supplement: Supplementary file 1 [file molecules-25-03936-s001.pdf]

Supplementary Materials

Article

Biochemical and Computational Approach of Selected Phytocompounds from *Tinospora crispa* in the Management of COVID-19

Ahmed Rakib [1], Arkajyoti Paul [2,3,4], Md. Nazim Uddin Chy [2,5], Saad Ahmed Sami [1], Sumit Kumar Baral [4], Mohuya Majumder [2], Abu Montakim Tareq [5], Mohammad Nurul Amin [6], Asif Shahriar [7], Md. Zia Uddin [3,8], Mycal Dutta [3,8], Trina Ekawati Tallei [9], Talha Bin Emran [3,*] and Jesus Simal-Gandara [10,*]

[1] Department of Pharmacy, Faculty of Biological Sciences, University of Chittagong, Chittagong 4331, Bangladesh; rakib.pharmacy.cu@gmail.com (A.R.); s.a.sami18pharm@gmail.com (S.A.S.)
[2] Drug Discovery, GUSTO A Research Group, Chittagong 4000, Bangladesh; arka.bgctub@gmail.com (A.P.); nazim107282@gmail.com (M.N.U.C.); mohuyamajumderbgctub@gmail.com (M.M.)
[3] Department of Pharmacy, BGC Trust University Bangladesh, Chittagong 4381, Bangladesh; zia@bgctub.ac.bd (M.Z.U.); mycal@bgctub.ac.bd (M.D.)
[4] Department of Microbiology, Jagannath University, Dhaka 1100, Bangladesh; akbaiub6@gmail.com
[5] Department of Pharmacy, International Islamic University Chittagong, Chittagong 4318, Bangladesh; montakim0.abu@gmail.com
[6] Department of Pharmacy, Atish Dipankar University of Science and Technology, Dhaka 1230, Bangladesh; amin.pharma07@gmail.com
[7] Department of Microbiology, Stamford University Bangladesh, 51 Siddeswari Road, Dhaka 1217, Bangladesh; abasifbl@gmail.com
[8] Department of Pharmacy, Jahangirnagar University, Savar, Dhaka 1342, Bangladesh
[9] Department of Biology, Faculty of Mathematics and Natural Sciences, Sam Ratulangi University, Manado 95115, Indonesia; trina_tallei@unsrat.ac.id
[10] Nutrition and Bromatology Group, Department of Analytical and Food Chemistry, Faculty of Food Science and Technology, University of Vigo–Ourense Campus, E32004 Ourense, Spain
* Correspondence: talhabmb@bgctub.ac.bd (T.B.E.); jsimal@uvigo.es (J.S.G.); Tel.: +88-01819-942214 (T.B.E.); +34-988-387000 (J.S.G.)

Figure Legends:

Figure S1. 2D and 3D interactions of Benzeneethanamine (A) and Camphenol (B) with the active site of SARS-CoV-2 M^{pro} (PDB ID: 6W63)

Figure S2. 2D and 3D interactions of Strophanthidin (C) and Retinal (D) with the active site of SARS-CoV-2 M^{pro} (PDB ID: 6W63)

Figure S3. 2D and 3D interactions of Trans-geranylgeraniol (E) and 3,4-Dihydroxymandelic acid (F) with the active site of SARS-CoV-2 M^{pro} (PDB ID: 6W63)

Figure S4. 2D and 3D interactions of Imidazolidin-4-one, 2-imino-1-(4-methoxy-6-dimethylamino-1,3,5-triazin-2-yl) (G) and Retinol (F) with the active site of SARS-CoV-2 M^{pro} (PDB ID: 6W63)

Figure S5. 2D and 3D interactions of alpha-Santalol (I) and Santalol, E-cis,epi-.beta.- (J) with the active site of SARS-CoV-2 M^{pro} (PDB ID: 6W63)

Figure S6. 2D and 3D interactions of spiro [4.5]dec-6-en-1-ol, 2,6,10,10-tetramethyl (K) and 3.beta.-Hydroxy-5-cholen-24-oic acid (L) with the active site of SARS-CoV-2 M^{pro} (PDB ID: 6W63)

Figure S7. 2D and 3D interactions of Phosphonoacetic acid, 3TMS derivative (M) and Nordazepam, TMS derivative (N) with the active site of SARS-CoV-2 M^{pro} (PDB ID: 6W63)

Figure S8. 2D and 3D interactions of 2,6-Dihydroxybenzoic acid, 3TMS derivative (O) and aR-Turmerone (P) with the active site of SARS-CoV-2 M^{pro} (PDB ID: 6W63)

Figure S9. 2D and 3D interactions of (Z)-.gamma.-Atlantone (Q) and Verbenyl angelate, cis- (R) with the active site of SARS-CoV-2 M^{pro} (PDB ID: 6W63)

Figure S10. 2D and 3D interactions of Tumerone (S) and Dibutyl phthalate (T) with the active site of SARS-CoV-2 M^{pro} (PDB ID: 6W63)

Figure S11. 2D and 3D interactions of (-)-Globulol (U) and Androstan-17-one, 3-ethyl-3-hydroxy-, (5.alpha) (V) with the active site of SARS-CoV-2 M^{pro} (PDB ID: 6W63)

Figure S12. 2D and 3D interactions of Yangambin (W) with the active site of SARS-CoV-2 M^{pro} (PDB ID: 6W63)

Figure S13. 2D and 3D interactions of Nelfinavir (X) and Lopinavir (Y) with the active site of SARS-CoV-2M^{pro} (PDB ID: 6W63).

FIGURE S1.

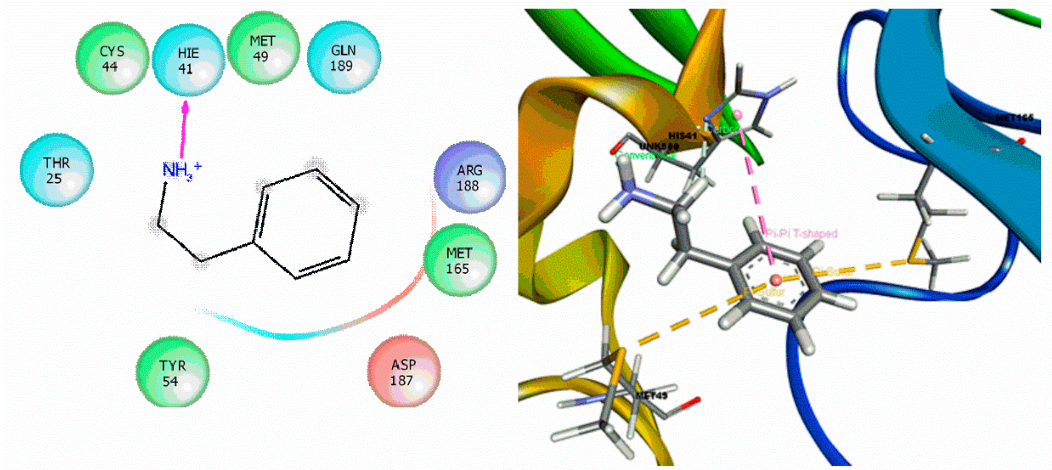
CYS 44
HIE 41
MET 49
GLN 189
THR 25
ARG 188
MET 165
TYR 54
ASP 187
NH3+

A

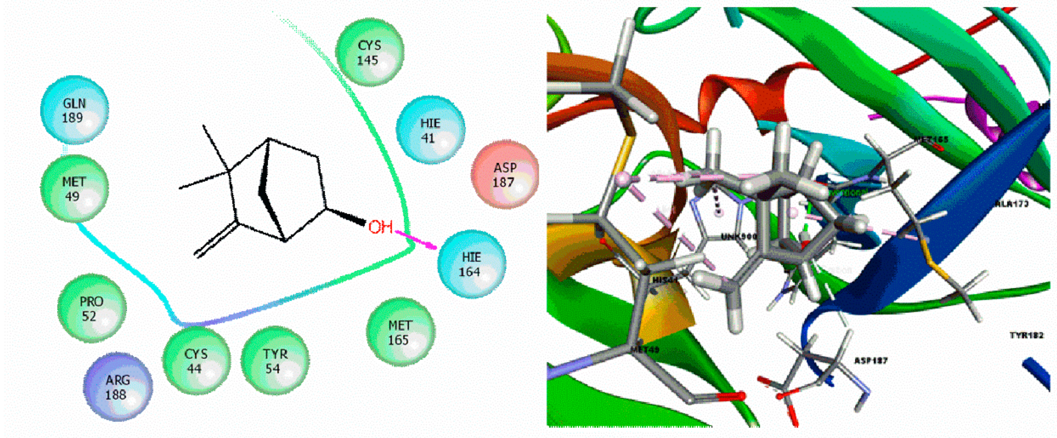
CYS 145
GLN 189
HIE 41
ASP 187
MET 49
OH
HIE 164
PRO 52
MET 165
CYS 44
TYR 54
ARG 188

B

FIGURE S2.

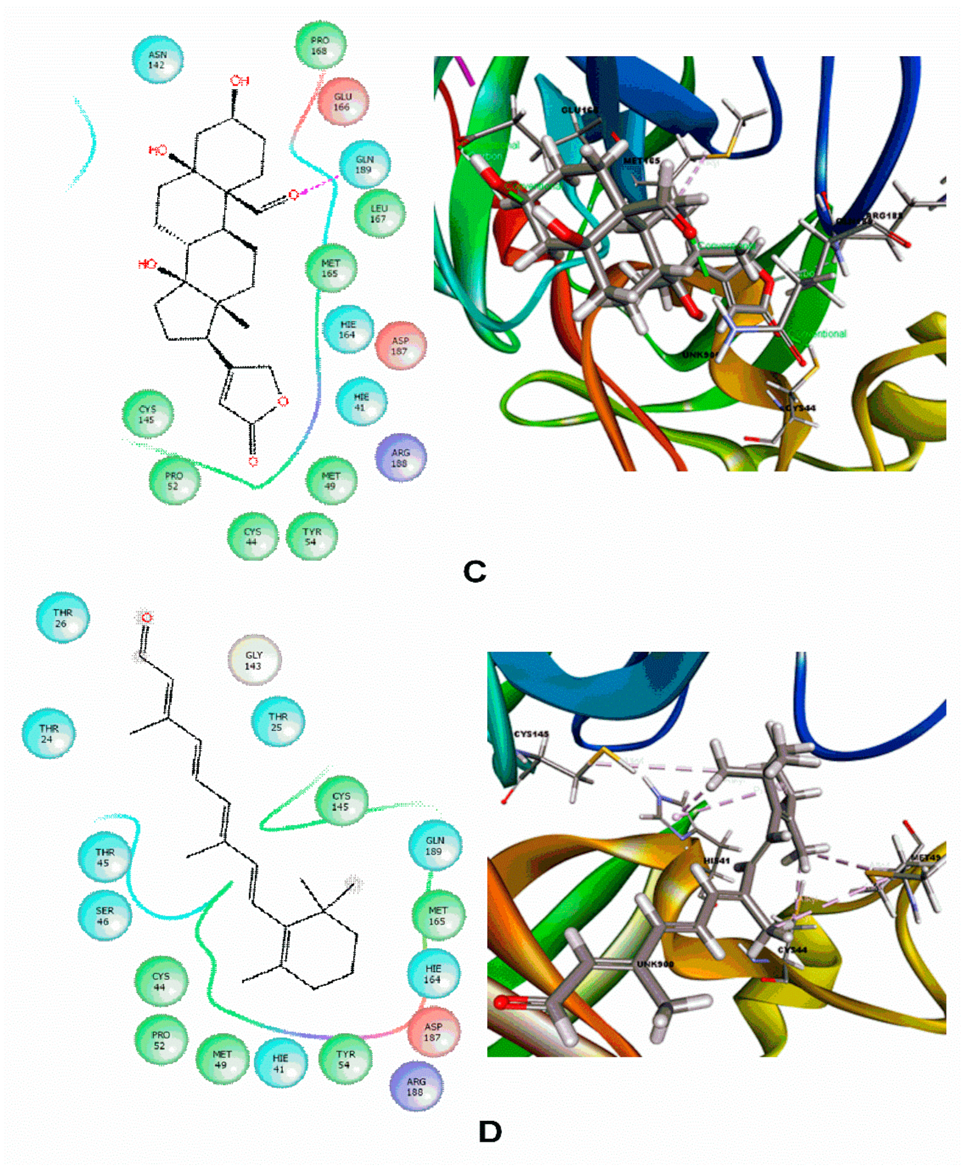
ASN 142
PRO 168
GLU 166
GLN 189
LEU 167
MET 165
HIE 164
ASP 187
HIE 41
ARG 188
CYS 145
PRO 52
MET 49
CYS 44
TYR 54
C
THR 26
GLY 143
THR 25
THR 24
CYS 145
THR 45
SER 46
GLN 189
MET 165
HIE 164
CYS 44
PRO 52
MET 49
HIE 41
TYR 54
ASP 187
ARG 188
D

FIGURE S3.

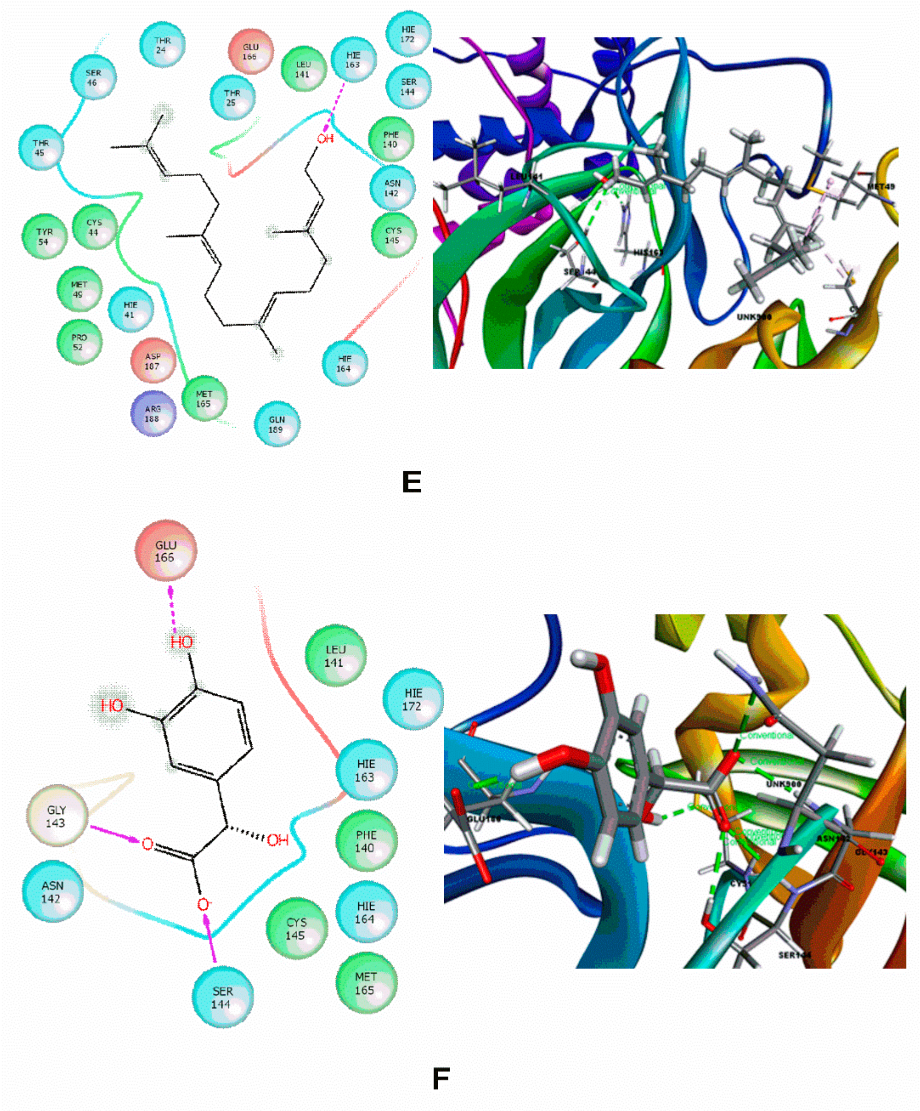
THR 24
GLU 166
LEU 141
HIE 163
HIE 172
SER 46
THR 25
SER 144
THR 45
PHE 140
OH
ASN 142
TYR 54
CYS 44
CYS 145
MET 49
HIE 41
PRO 52
ASP 187
HIE 164
MET 165
ARG 188
GLN 189
E
GLU 166
HO
LEU 141
HIE 172
HO
HIE 163
GLY 143
O
OH
PHE 140
ASN 142
O-
CYS 145
HIE 164
SER 144
MET 165
F

FIGURE S4.

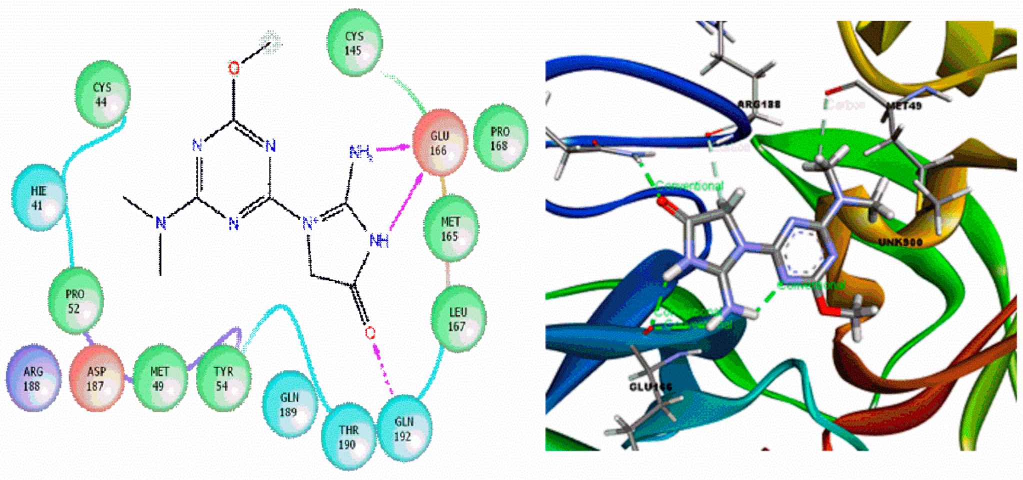

CYS 145
CYS 44
GLU 166
PRO 168
HIE 41
MET 165
PRO 52
LEU 167
ARG 188
ASP 187
MET 49
TYR 54
GLN 189
THR 190
GLN 192
ARG188
MET49
UNK900
GLU166

G

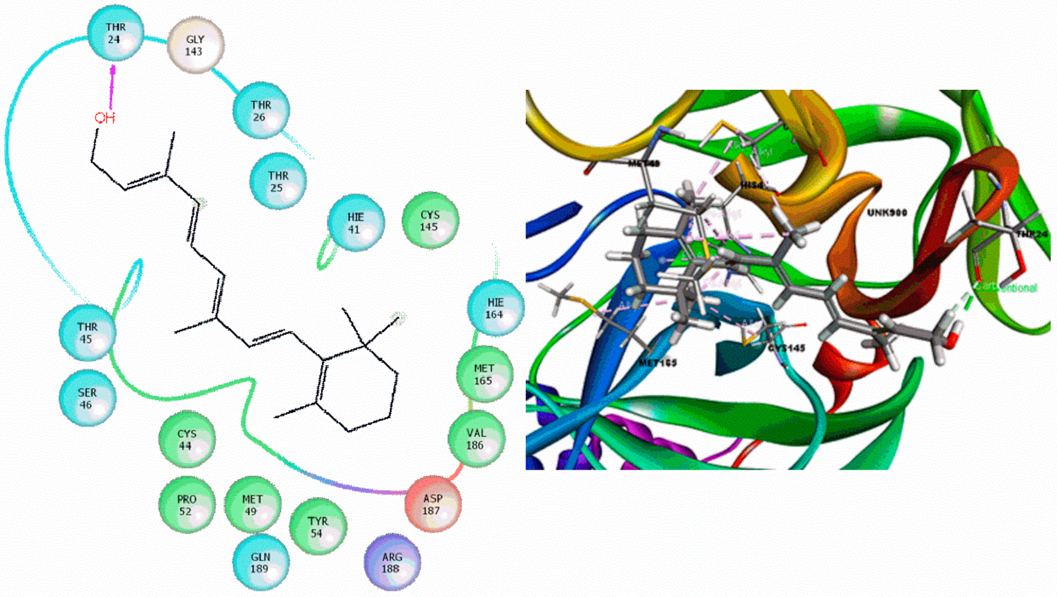

THR 24
GLY 143
THR 26
THR 25
HIE 41
CYS 145
HIE 164
THR 45
MET 165
SER 46
VAL 186
CYS 44
PRO 52
MET 49
TYR 54
ASP 187
GLN 189
ARG 188
UNK900
MET165
CYS145

H

FIGURE S5.

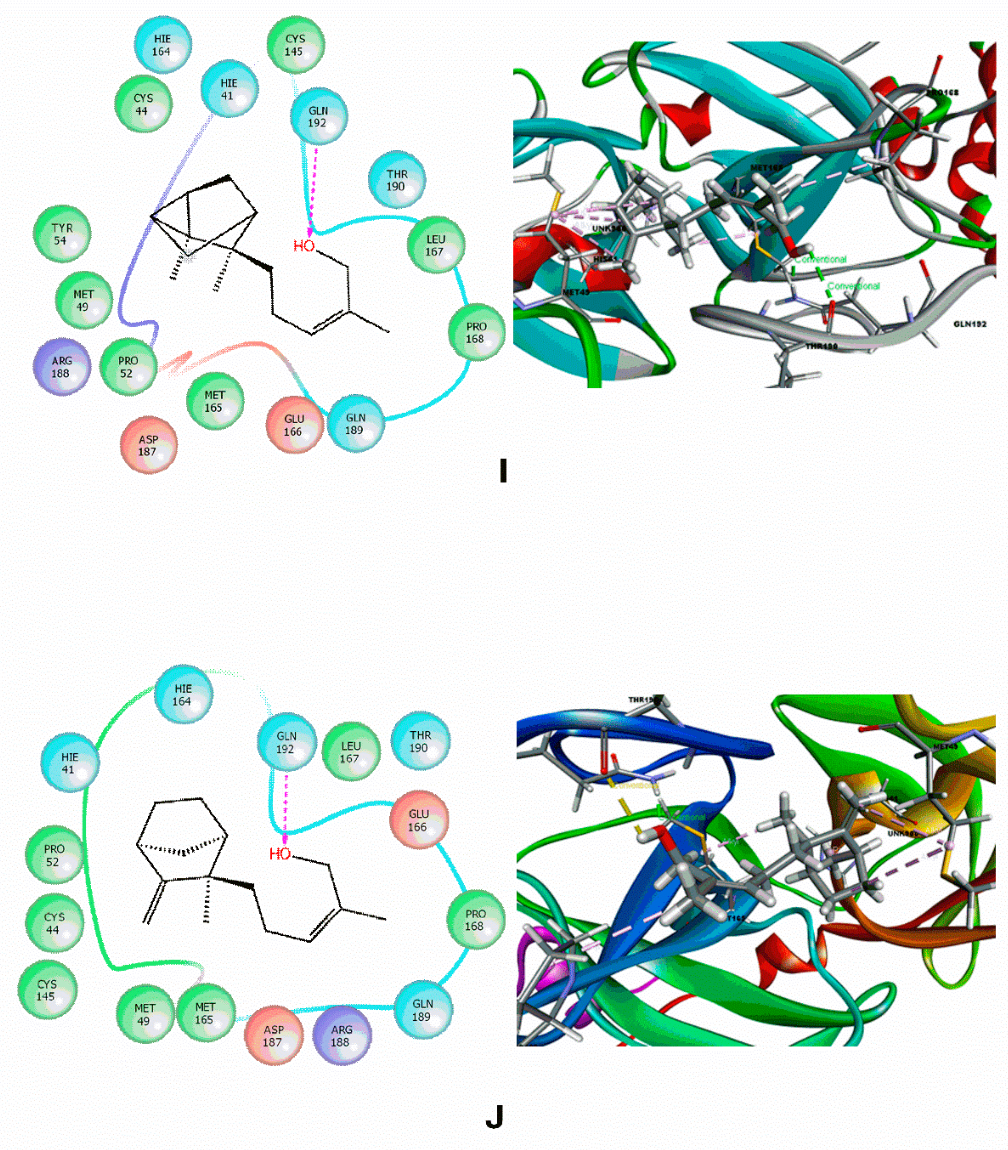
HIE 164
CYS 145
HIE 41
CYS 44
GLN 192
THR 190
TYR 54
HO
LEU 167
MET 49
PRO 168
ARG 188
PRO 52
MET 165
GLU 166
GLN 189
ASP 187
I
HIE 164
GLN 192
LEU 167
THR 190
HIE 41
GLU 166
HO
PRO 52
PRO 168
CYS 44
CYS 145
MET 49
MET 165
ASP 187
ARG 188
GLN 189
J

FIGURE S6.

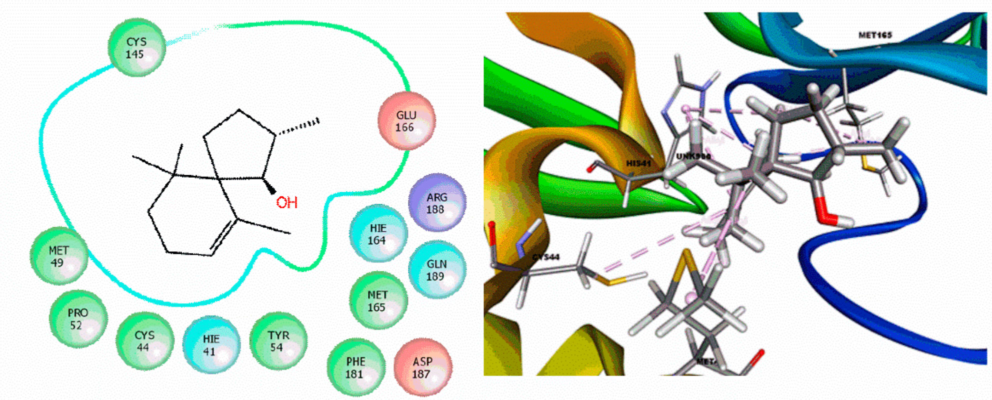
CYS 145
GLU 166
OH
ARG 188
HIE 164
GLN 189
MET 49
MET 165
PRO 52
CYS 44
HIE 41
TYR 54
PHE 181
ASP 187
MET165
HIS41
UNK900
CYS44

K

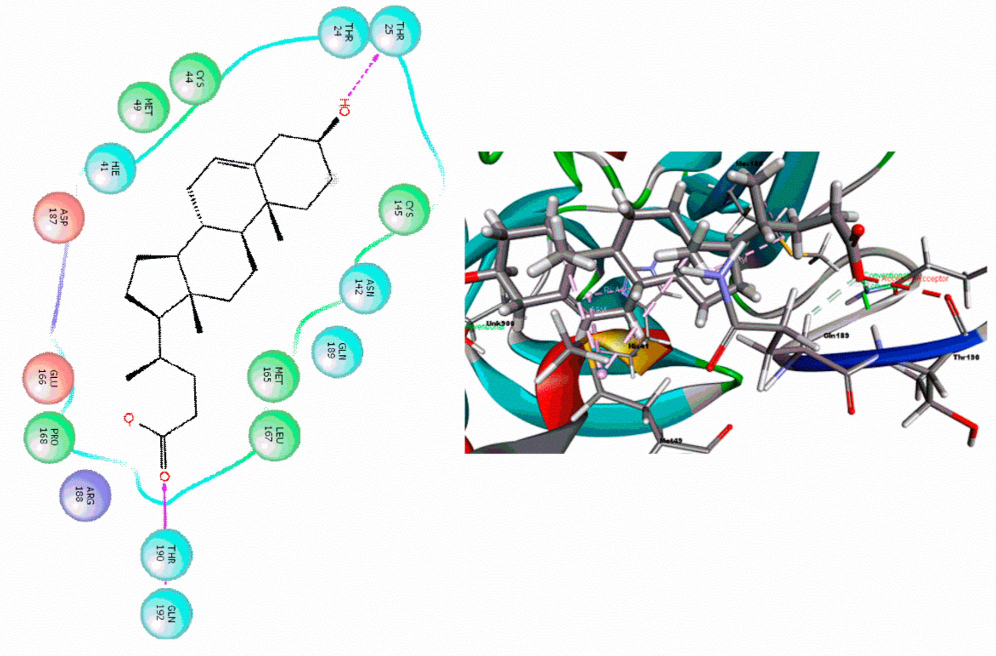
THR 24
THR 25
CYS 44
MET 49
HO
HIE 41
ASP 187
CYS 145
ASN 142
GLN 189
GLU 166
MET 165
PRO 168
LEU 167
O
ARG 188
THR 190
GLN 192

FIGURE S7.

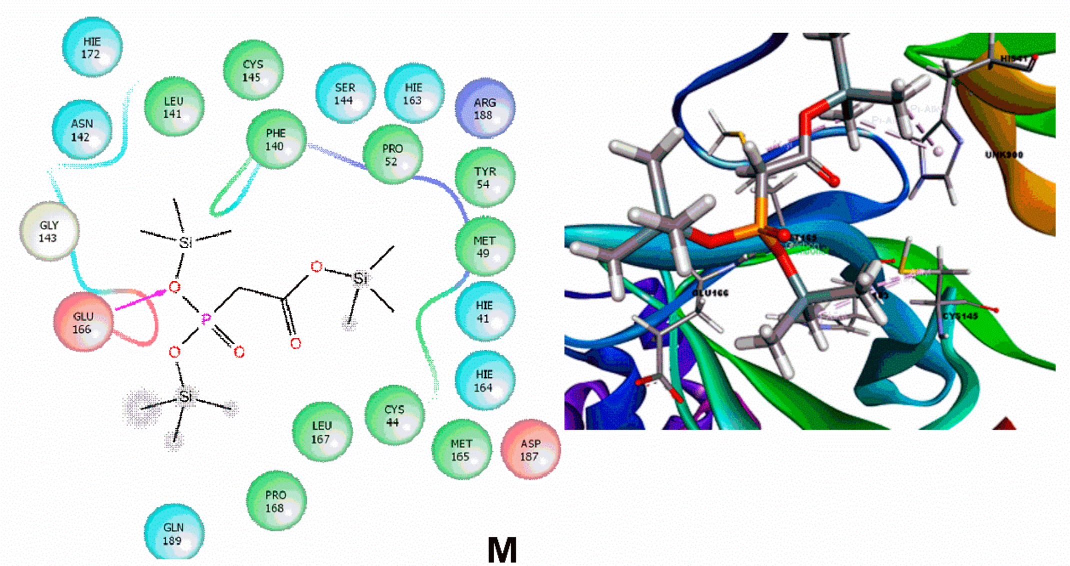
HIE 172
ASN 142
LEU 141
CYS 145
PHE 140
SER 144
HIE 163
ARG 188
PRO 52
TYR 54
GLY 143
MET 49
GLU 166
HIE 41
HIE 164
CYS 44
LEU 167
MET 165
ASP 187
PRO 168
GLN 189

M

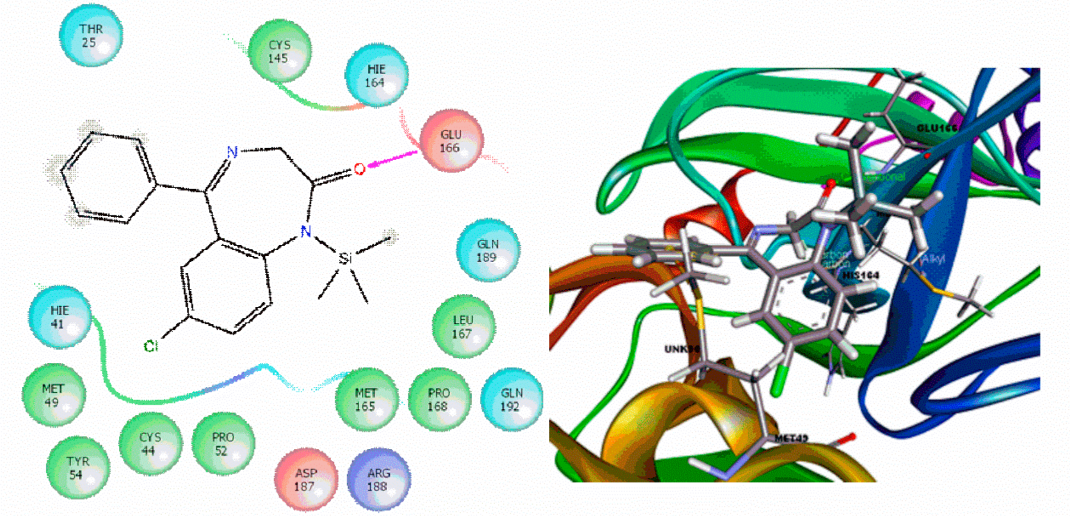
THR 25
CYS 145
HIE 164
GLU 166
GLN 189
HIE 41
LEU 167
MET 49
MET 165
PRO 168
GLN 192
CYS 44
PRO 52
TYR 54
ASP 187
ARG 188

N

FIGURE S8.

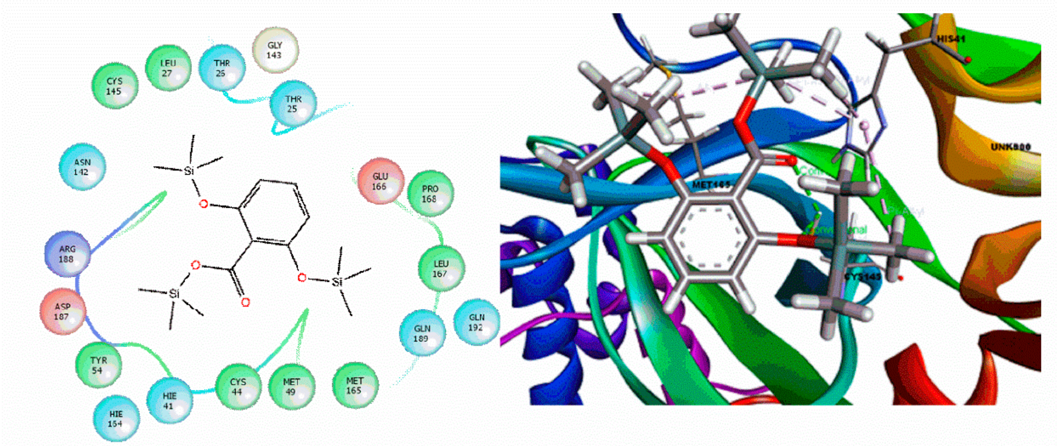

CYS 145
LEU 27
THR 26
GLY 143
THR 25
ASN 142
ARG 188
ASP 187
TYR 54
HIE 41
CYS 44
MET 49
MET 165
GLU 166
PRO 168
LEU 167
GLN 189
GLN 192
MET165

O

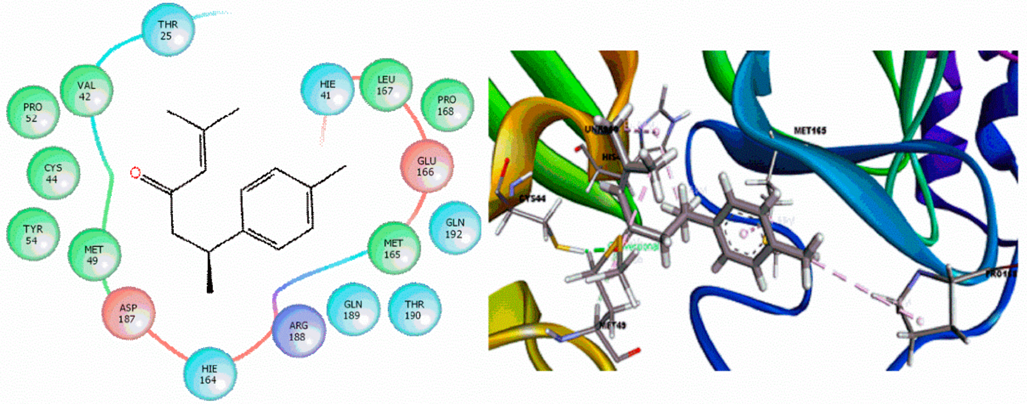

THR 25
VAL 42
PRO 52
CYS 44
TYR 54
MET 49
ASP 187
HIE 164
ARG 188
GLN 189
THR 190
MET 165
GLN 192
GLU 166
PRO 168
LEU 167
HIE 41
MET165

P

FIGURE S9.

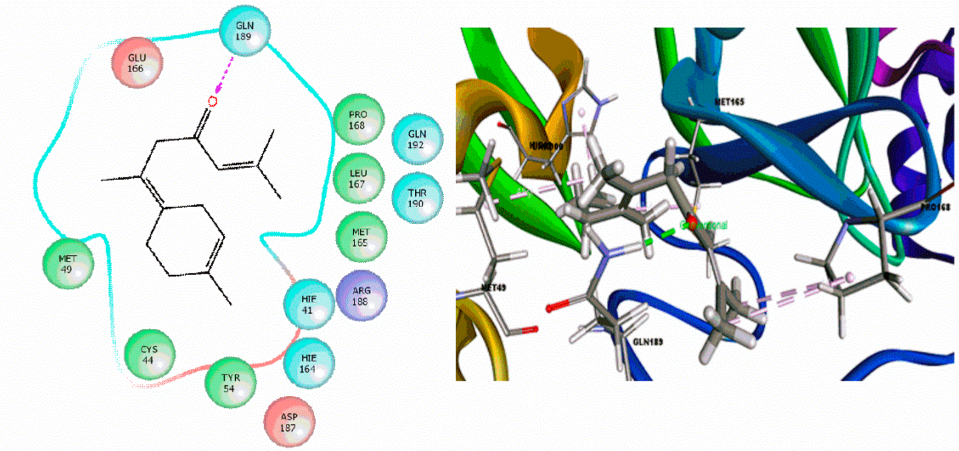
GLN 189
GLU 166
PRO 168
GLN 192
LEU 167
THR 190
MET 165
MET 49
HIE 41
ARG 188
CYS 44
HIE 164
TYR 54
ASP 187
MET165
PRO168
MET49
GLN189

Q

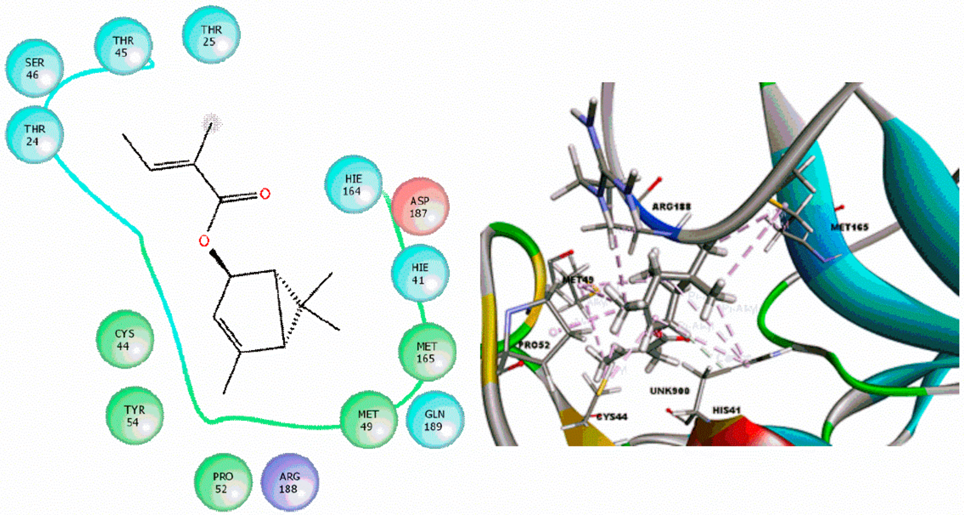
THR 45
THR 25
SER 46
THR 24
HIE 164
ASP 187
HIE 41
CYS 44
MET 165
TYR 54
MET 49
GLN 189
PRO 52
ARG 188
ARG188
MET165
MET49
PRO52
UNK900
HIS41
CYS44

R

FIGURE S10.

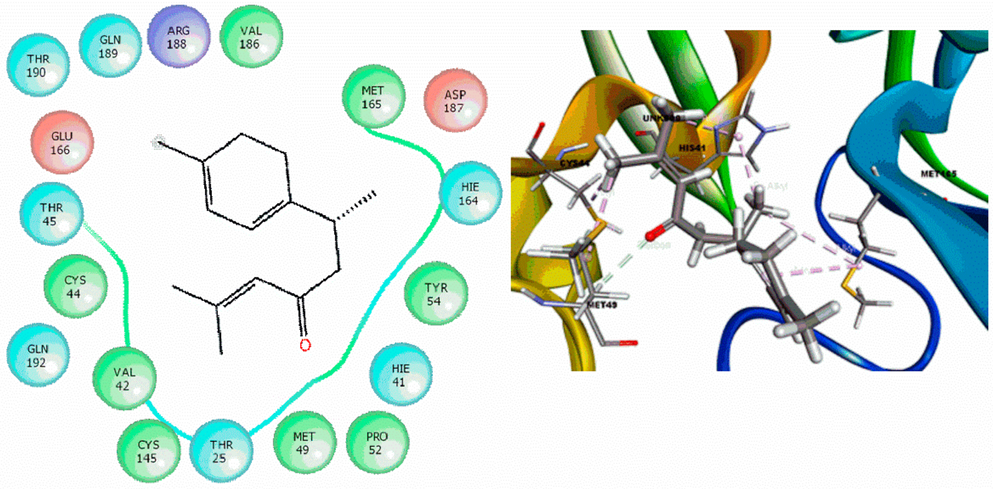

S

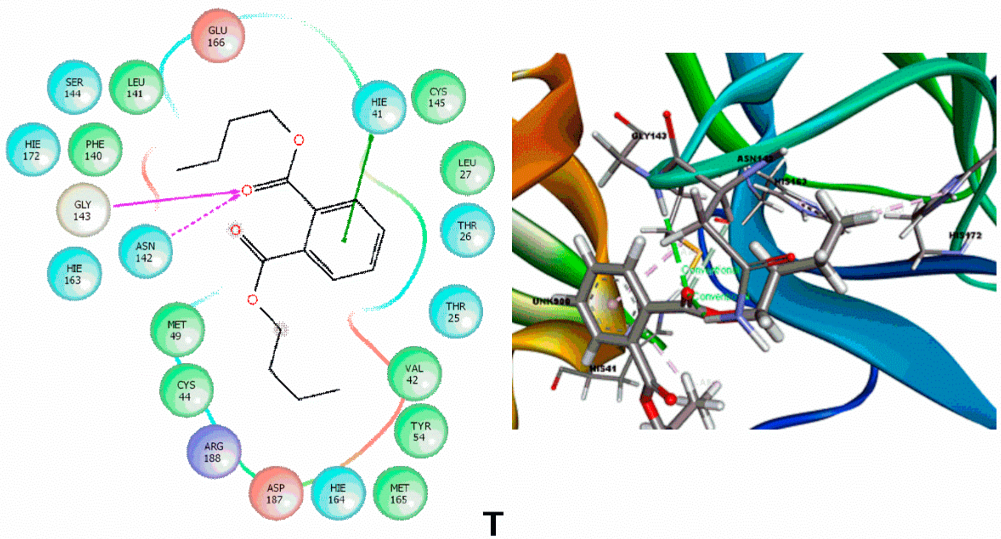

T

FIGURE S11.

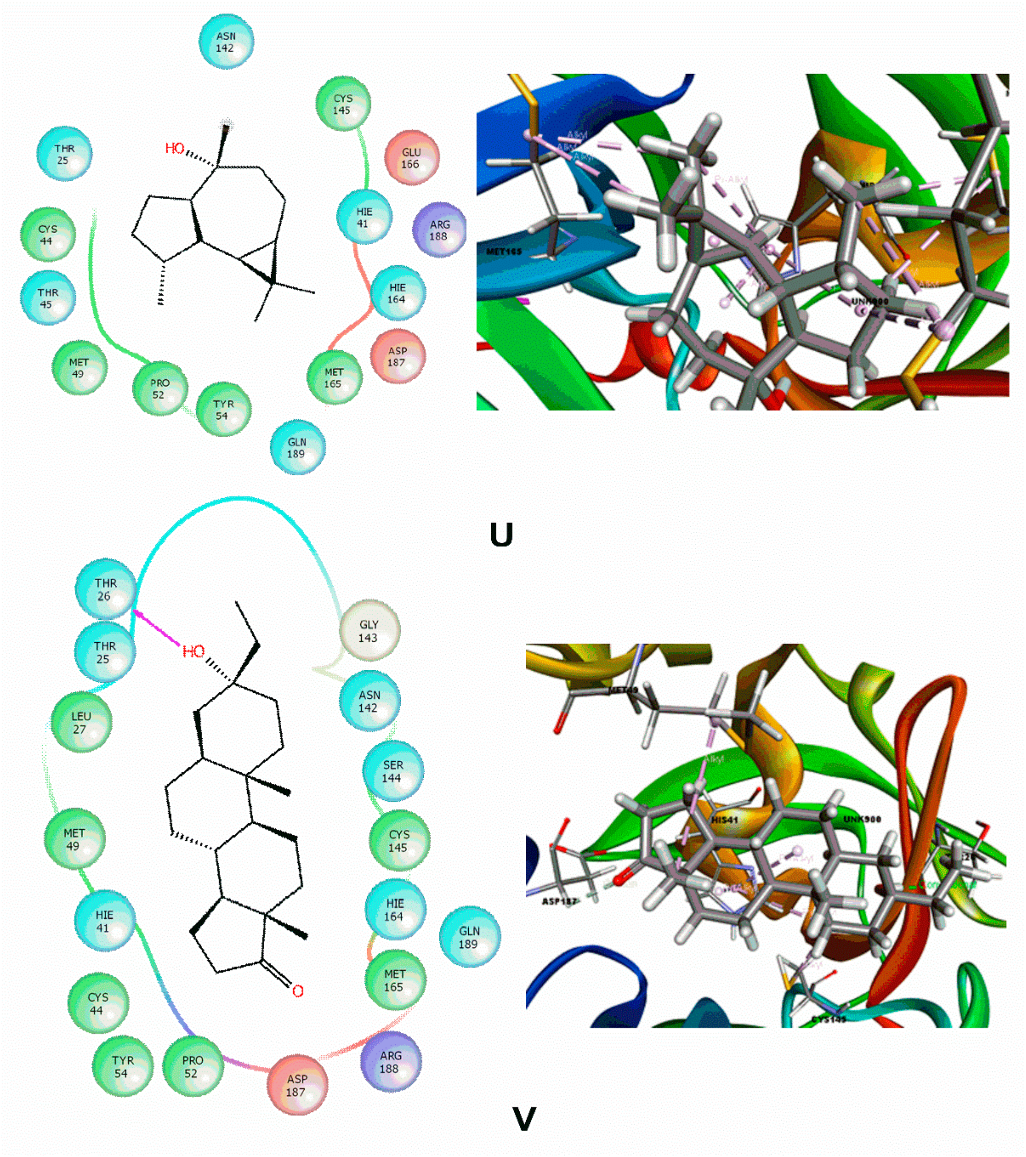
ASN 142
CYS 145
THR 25
GLU 166
HO
CYS 44
HIE 41
ARG 188
THR 45
HIE 164
ASP 187
MET 49
MET 165
PRO 52
TYR 54
GLN 189
U
THR 26
GLY 143
THR 25
HO
ASN 142
LEU 27
SER 144
MET 49
CYS 145
HIE 41
HIE 164
GLN 189
O
MET 165
CYS 44
TYR 54
PRO 52
ASP 187
ARG 188
V

FIGURE S12.

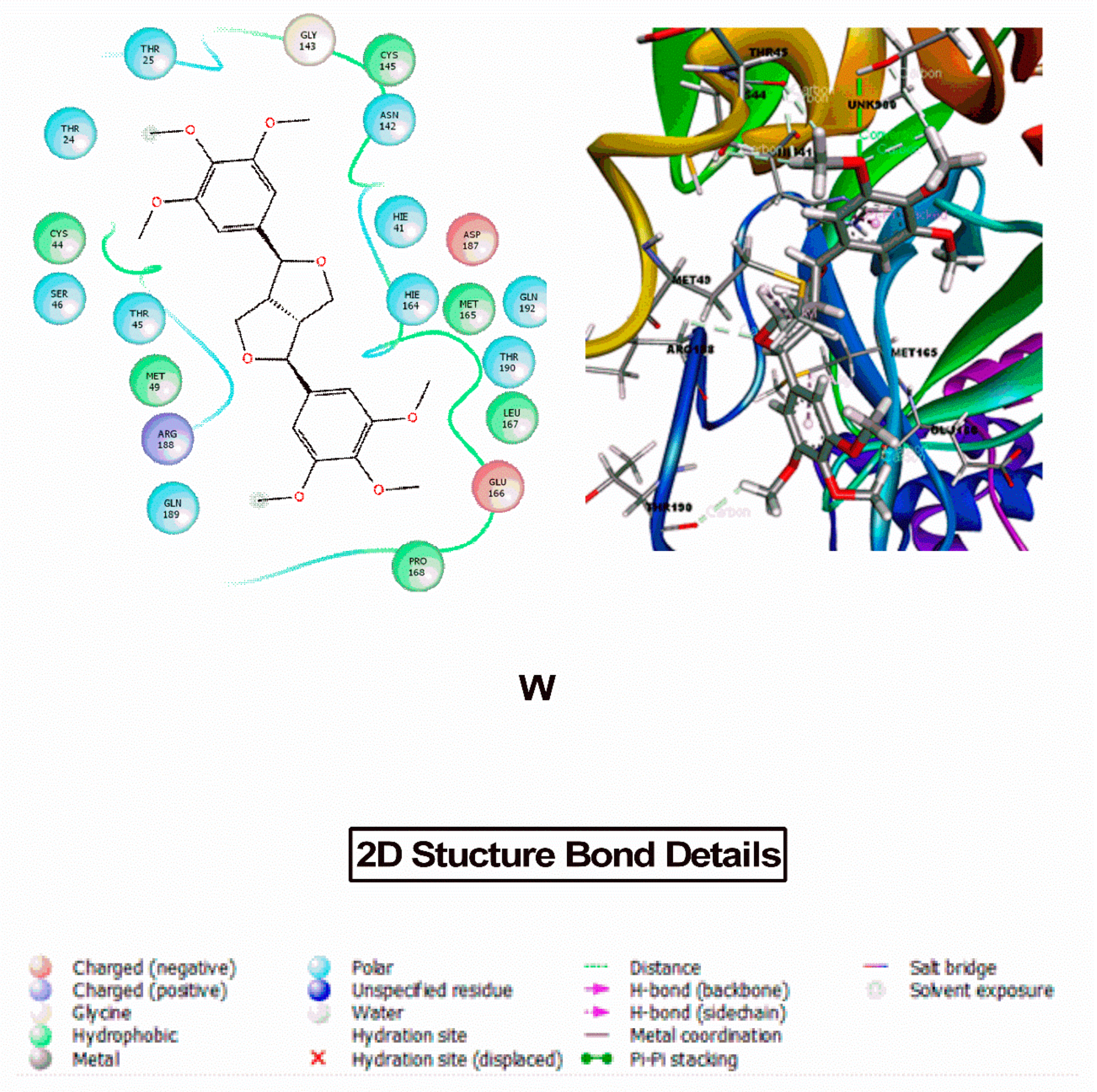
THR 25
GLY 143
CYS 145
THR 24
ASN 142
HIE 41
ASP 187
CYS 44
SER 46
THR 45
HIE 164
MET 165
GLN 192
THR 190
MET 49
LEU 167
ARG 188
GLU 166
GLN 189
PRO 168
THR45
UNK900
MET49
ARG188
MET165
THR190
W
2D Stucture Bond Details
Charged (negative)
Charged (positive)
Glycine
Hydrophobic
Metal
Polar
Unspecified residue
Water
Hydration site
Hydration site (displaced)
Distance
H-bond (backbone)
H-bond (sidechain)
Metal coordination
Pi-Pi stacking
Salt bridge
Solvent exposure

FIGURE S13.

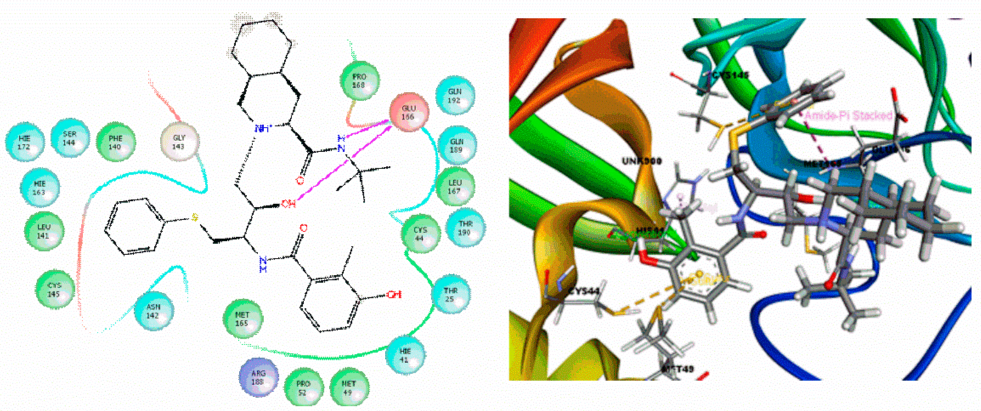

X

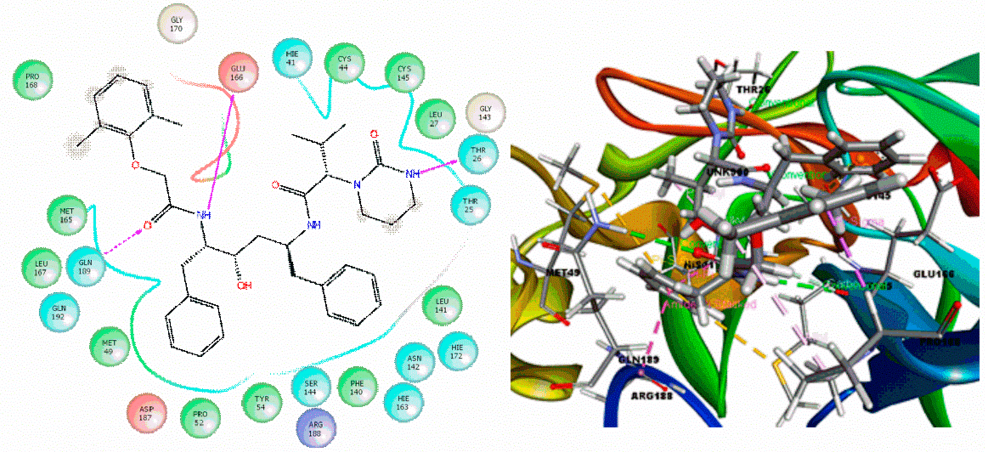

Y
